# Enantioselective Recognition of Racemic Amino Alcohols in Aqueous Solution by Chiral Metal‐Oxide Keplerate {Mo_132_} Cluster Capsules

**DOI:** 10.1002/chem.202100899

**Published:** 2021-07-30

**Authors:** Robert W. Pow, Zoё L. Sinclair, Nicola L. Bell, Nancy Watfa, Yousef M. Abul‐Haija, De‐Liang Long, Leroy Cronin

**Affiliations:** ^1^ Department of Chemistry University of Glasgow University Avenue Glasgow G12 8QQ UK

**Keywords:** amino alcohols, chiral recognition, host-guest systems, NMR spectroscopy, polyoxometalates

## Abstract

Determining the relative configuration or enantiomeric excess of a substance may be achieved using NMR spectroscopy by employing chiral shift reagents (CSRs). Such reagents interact noncovalently with the chiral solute, resulting in each chiral form experiencing different magnetic anisotropy; this is then reflected in their NMR spectra. The Keplerate polyoxometalate (POM) is a molybdenum‐based, water‐soluble, discrete inorganic structure with a pore‐accessible inner cavity, decorated by differentiable ligands. Through ligand exchange from the self‐assembled nanostructure, a set of chiral Keplerate host molecules has been synthesised. By exploiting the interactions of analyte molecules at the surface pores, the relative configuration of chiral amino alcohol guests (phenylalaninol and 2‐amino‐1‐phenylethanol) in aqueous solvent was establish and their enantiomeric excess was determined by ^1^H NMR using shifts of ΔΔ*δ*=0.06 ppm. The use of POMs as chiral shift reagents represents an application of a class that is yet to be well established and opens avenues into aqueous host‐guest chemistry with self‐assembled recognition agents.

## Graphical abstract

**Chiral resolution** of racemic amino alcohol guests (phenylalaninol and 2‐amino‐1‐phenylethanol) has been achieved by using a chirally coordinated enantiopure polyoxometalate host, {Mo_132_(lactate)_30_}. NMR spectroscopic methods, including DOSY measurements, were applied to determine the enantiomeric excesses of guest mixtures in addition to the association constants of the host‐guest system binding process.

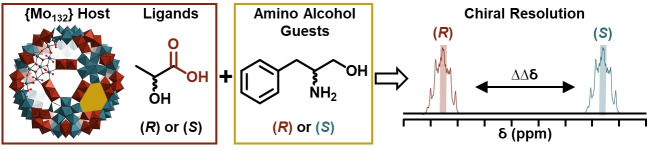

## Introduction

Chiral shift reagents (CSRs) or chiral solvating agents (CSAs) allow the relative configuration or enantiomeric excess of a mixture of chiral molecules to be determined by NMR through the formation of noncovalent diastereomers. This is important as the specific stereochemistry of a molecule determines the chemical, physical and biological properties of the species. Structures including cyclodextrins,^[1]^ calixarenes, crown ethers, porphyrins,^[2]^ cucurbiturils,^[3]^ and cages/cavities,[^4^, ^5^, ^6^, ^7^] have been explored as hosts for probing chiral analytes by noncovalent interactions using solution‐state NMR, mostly in organic media. The development of extended chiral metal‐organic frameworks and their application to NMR chiral recognition methods has thus far been limited to solid‐state analyses.[^8^, ^9^, ^10^, ^11^] Despite this, the use of discrete inorganic hosts, such as polyoxometalates (POMs), for the same role has not yet been achieved. These chiral inorganic nanostructures can be separated into two general classes, 1) purely inorganic molecules and 2) inorganic‐organic hybrid structures, with the latter possessing chirality in either the inorganic, the organic component, or both.[^12^, ^13^] Both systems may be soluble in water and, depending upon the countercation, in nonaqueous media.

**Figure 1:**
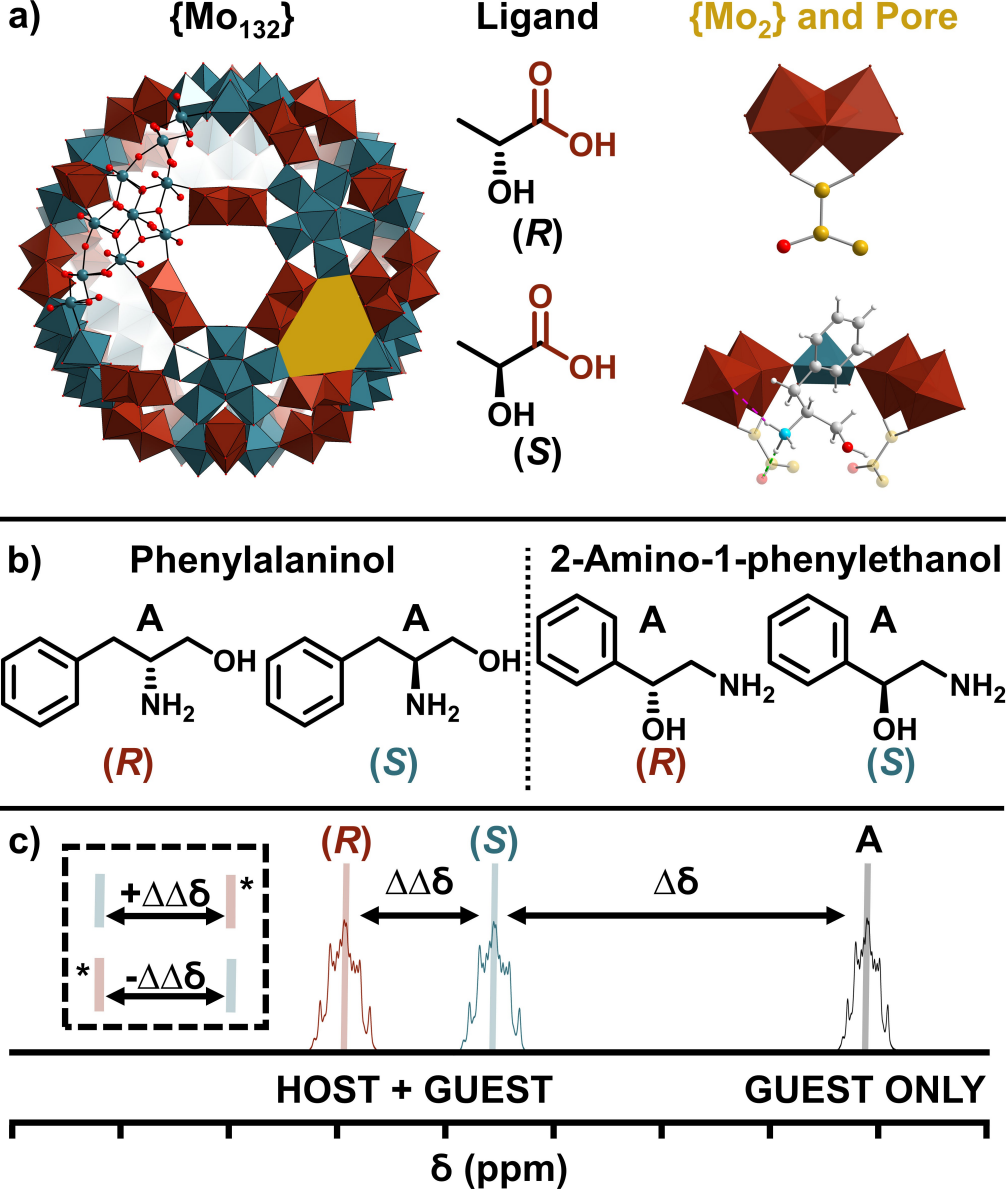
Overall representation of the work presented. a) Structural framework of {Mo_132_}, showing the building block polyhedra ({Mo_2_}: red, and {(Mo)Mo_5_}: blue) in addition to the metal‐oxo bonding in ball‐and‐stick representation (Mo atoms: blue, O: red). One of the 20 {Mo_2_}‐formed surface pores is highlighted in yellow. The chiral ligands used throughout this work (C atoms: yellow) showing the binding between the {Mo_2_} linkers and these carboxylate ligands. A model of an individual pore with (*S*)‐phenylalaninol (N atom: turquoise, O atom: red) is shown and the noncovalent interactions it has with the {Mo_2_} and ligands. Shown from above and side on. b) The isomers of the amino alcohol guests separated here, and c) the general schematic of spectral assignment used herein (not to scale).

The molybdenum‐based spherical {Mo_132_} Keplerate‐type structure provides an interesting inorganic framework for use in the study of host‐guest interactions (Figure 1a).^[14]^ The hollow character of the structure offers a distinct enclosed environment within which entrapped species are exposed to an alternative chemical environment to those found in the bulk media. This confined cavity (volume ca 1.5 nm^3^) acts as a container for regioselective reactions to occur, and has the ability to separate and stabilise entrapped species.[^15^, ^16^, ^17^] This internal cavity is accessible through twenty surface pores (Figure 1a, yellow), whose rigid nature allows for selective uptake of guest species based on size. Further, each of the surface pores offers a secondary site for guest interaction through electrostatic interactions, resulting in trapping of cationic species and affording the molecule with polytopic receptor properties.[^17^, ^18^] The size of trapped species range from small cations, such as Na^+^, to molecules such as guanidinium.[^18^, ^19^, ^20^] The positions of the ions at the pores (ca 0.3 nm diameter, C_3*V*_ local symmetry) are dictated by the size of the species, leading to these structures being described as “nano‐ion chromatographs”.

Herein, we describe the synthesis and characterisation of chiral {Mo_132_} structures, [Mo_132_O_372_(H_2_O)_72_(L)_30_] (where L: (*R*)/(*S*)‐lactate) possessing chiral internal cavities facilitated by the affixation of chiral ligands, referred to generally as {Mo_132_(lactate)_30_} (Figure 1a). We have subsequently demonstrated its noncovalent chiral recognition towards amino alcohol species in aqueous solutions, including the assignment of relative configurations of guest species and the determination of approximate enantiomeric excesses of scalemic mixtures.

## Results and Discussion

### Structure characterisation: Solid‐ and solution‐state methods

The enantiomerically pure {Mo_132_} host structures were synthesised by adding an excess of the relevant ligand species (>100 equiv. cf. {Mo_132_}) to an aqueous solution of {Mo_132_(SO_4_)_30_}. Single crystals, suitable for X‐ray crystallographic analysis, were grown by slow evaporation of the solvent over five days. Although the ligand exchange process is generally straightforward, in this instance more careful control of pH was required (pH 2.8) as pH values outside a narrow range caused the formation of precipitate or poor crystal quality. Single‐crystal X‐ray structure analyses reveal that the typical spherical {Mo_132_} framework is retained for all products. This molybdate framework can be regarded as an icosahedron in which twelve {(Mo^VI^)Mo^VI^_5_O_21_(H_2_O)_6_} pentagonal units are placed at the vertices and are linked by thirty {Mo^V^_2_O_4_}^2+^ dinuclear linkers (Figure 1a, blue and red polyhedra, respectively). The relevant ligands coordinate at these {Mo^V^_2_O_4_}^2+^ linkers via their carboxylate functional groups, with their tails hanging towards the centre of the internal cavity. Despite the analytical data confirming the intactness of the ligands after exchange, the full organic “tails” could not be fully resolved from the diffraction data due to the high absorption by the electron‐dense Mo framework and the free rotation of the ligand tails within the POM inner cavity. Both factors increase the disorder in the positions of the ligands beyond the carboxylate atoms. This difficulty in resolving ligands beyond the carboxylate moiety within the {Mo_132_}‐framework is well known and is found in many previously reported crystal structures.[^21^, ^22^] Due to the high symmetry of the Mo framework (*I*_h_), all structures crystallise in the *R*‐3 centrosymmetric space group, rather than a chiral space group.

The ^1^H NMR and ^13^C NMR spectra of {Mo_132_((*R*)‐lactate)_30_} displays two sets of well‐resolved signals corresponding to free (*R*)‐lactic acid and coordinated (*R*)‐lactate ligands. Although the ligands are found in two positions upon dissolution, the majority of ligands (approximately 26/30) are retained within the cavity. The broad signals belonging to encapsulated ligands are found upfield (2.9 ppm (CH(1)) and 0.1 ppm (CH_3_(2), respectively) with respect to the corresponding sharp signals of the free lactic acid species (Figure S5 in the Supporting Information). The two signal sets, which are rapidly established, indicate that the ligand exchange process which is initiated upon crystal dissolution is slow on the timescale of the NMR measurements. The coordinated ligands are replaced by water ligands, as has been previously reported.^[21]^ The resulting equilibrium is stable under static conditions over a timescale of at least several weeks, as indicated by unchanging peak integrals. This assignment of the two domains of the species was supported by ^1^H DOSY NMR where the diffusion coefficient decreased from 520 pm^2^ s^−1^ for the solvated molecules to 111 pm^2^ s^−1^ for the coordinated (*R*)‐lactate ligands within {Mo_132_} (Figure S8). The presence of both encapsulated and free, solvated lactates demonstrates that the {Mo_132_} framework is still intact in the solution and a proportion of the lactates are retained inside the cavity, available as chiral centres for enantioselective recognition. Additionally, the simultaneous existence of both peak sets demonstrates that the ligands are in a slow exchange system between their solvated free state and their coordinated, encapsulated state. To confirm our assignment of the ^1^H NMR spectra, HSQC NMR was performed. Typical broad and sharp peaks related to the coordinated and solvated lactate ligands are observed, respectively, at: [^1^H_FREE_/^1^H_ENCAPSULATED_:^13^C] (CH(1):C‐β) 3.9/2.8 ppm: 69 ppm, and (CH_3_(2):C‐γ) 1.2 ppm/0.0 ppm: 19 ppm (Figure S7).

Although the ligand structure could not be resolved in the solid‐state, we wanted to confirm that the stereostable ligands had indeed retained their stereochemistry upon their coordination within the {Mo_132_} structure. The presence of (*R*)‐ or (*S*)‐lactate leads to a CD response of their respective structures upon crystal dissolution. The obtained spectra of {Mo_132_((*R*)‐lactate)_30_} and {Mo_132_((*S*)‐lactate)_30_} display opposite ellipticity of one another, with peaks centred at 214 nm originating from the lactate ligands, demonstrating the clusters’ opposing optical activities as a result of the coordinated enantiopure ligands (Figure S1). Compared with free lactate, there are slight differences in the CD signals, with absorption effects of the {Mo_132_} framework perturbing the obtained spectra, however the opposing ellipticity of the spectra indicates that the configuration of lactate is preserved upon coordination to {Mo_132_}. The CD signal corresponding to {Mo_132_} is, however, not detected at higher wavelengths due to the rather strong adsorption arising from intervalence charge‐transfer between Mo^V^ and Mo^VI^ centres in {Mo_132_}, which greatly suppresses the CD response transferred by the lactate ligands.

Finally, the IR spectra of the {Mo_132_(lactate)_30_} structures were used to confirm the complete replacement of the sulfate ligands (Figure S4). The characteristic triplet pattern, due to the *v*_3_ stretching mode of the bidentate SO_4_^2−^ ligands (C_2v_) in the 1030–1190 cm^−1^ range, is only present in the {Mo_132_(SO_4_)_30_} spectrum and is not observed in the {Mo_132_(lactate)_30_} spectra, thus indicating that the replacement of these ligands has been achieved.

### Application of {Mo_132_(lactate)_30_} structures as chiral shift reagents

After confirming the configuration and stability of the chirally decorated {Mo_132_} structures in solution, we examined the extent of their chiral recognition of enantiopure guests. We selected amino alcohol guests due to their favorable solubility in aqueous solution, which our {Mo_132_} POM host is readily soluble in, and, as their amine group is positively charged at the pH studied here (ca pH 3), primary interaction at the {Mo_2_}‐formed surface pores was anticipated. In addition, a lack of coordination donors precludes the guest from replacing coordinated ligands of the {Mo_132_} host. The preferred affinity of positively charged species to the surface pores has been previously shown by Cadot et al.,^[23]^ while the effects of cation size at these sites have been described by Müller et al., highlighting that cationic species can be found deeper (closer to the internal cavity) or shallower in the pore depending upon the ionic size.^[18]^

Amino alcohols have uses in industrial and pharmacological applications. Enantiopurity of amino alcohols is important in this context as (*S*)‐propranolol is an active beta‐blocker, whereas the (*R)‐* isomer is ineffective in this application but has utility as a potential contraceptive.^[24]^ A universal noncovalent chiral shift reagent for the determination of relative configuration by NMR spectroscopy does not exist and the chiral recognition of amino alcohols has been achieved using several chiral shift reagents, including arylcarboxylic acids,[^25^, ^26^, ^27^] atropisomers,^[28]^ calixarenes/resorcinarenes,[^29^, ^30^, ^31^, ^32^, ^33^, ^34^, ^35^, ^36^] cyclodextrins,^[37]^ crown ethers,[^38^, ^39^, ^40^] phosphorous‐containing reagents,[^41^, ^42^, ^43^, ^44^] and metallocomplexes,[^45^, ^46^] amongst others.[^47^, ^48^, ^49^, ^50^] The separation of amino alcohols has been almost exclusively limited to organic solvents except for resorcinarene and cyclodextrin hosts which have displayed separation in aqueous media.

Introducing 12 equivalents of the phenylalaninol guest species to a 5 mM D_2_O solution of {Mo_132_((*R*)‐lactate)_30_} resulted in the presence of an additional single set of sharp peaks in the ^1^H NMR spectrum. These sharp peaks, which were downfield shifted in comparison to the spectrum of the amino alcohol in D_2_O only, indicate that the interaction of the guest with the {Mo_132_} structure occurs under a fast‐exchange regime, in contrast to the two‐set slow‐exchange mechanism exhibited by the ligand species, described previously. Twelve equivalents of guest per host (i. e. 0.6 equiv. guest/pore, as each {Mo_132_} host contains 20 individual pores) was chosen for NMR study after titrations indicated that the peak profile of the guests remained unchanged up to and including this number of equivalents, except for increasing guest peak intensities. Beyond this value the peaks broadened and the resulting solution began to form precipitate due to the initial formation of a surfactant encapsulated cluster (SEC; Figure S19).^[51]^

Any peak shifts of the guest isomers, from their positions in D_2_O only, are denoted Δ*δ* (Figure 1c). To remove ambiguity in the discussion of peak positions and relative shifts, the extent of shift separation between magnetically inequivalent guest peaks (ΔΔ*δ*) will be given for the guest *R* isomer cf. the *S* isomer. For example, a shift for an *R* guest that is further downfield than the *S* form will be given a negative value (−ΔΔ*δ*), and where the shift separation is reversed, with the (*R*) isomer more upfield in comparison to the (*S*)‐guest isomer, the separation will be noted with a positive sign (+ΔΔ*δ*).

### Phenylalaninol guests with {Mo_132_(lactate)_30_} hosts

**Figure 2:**
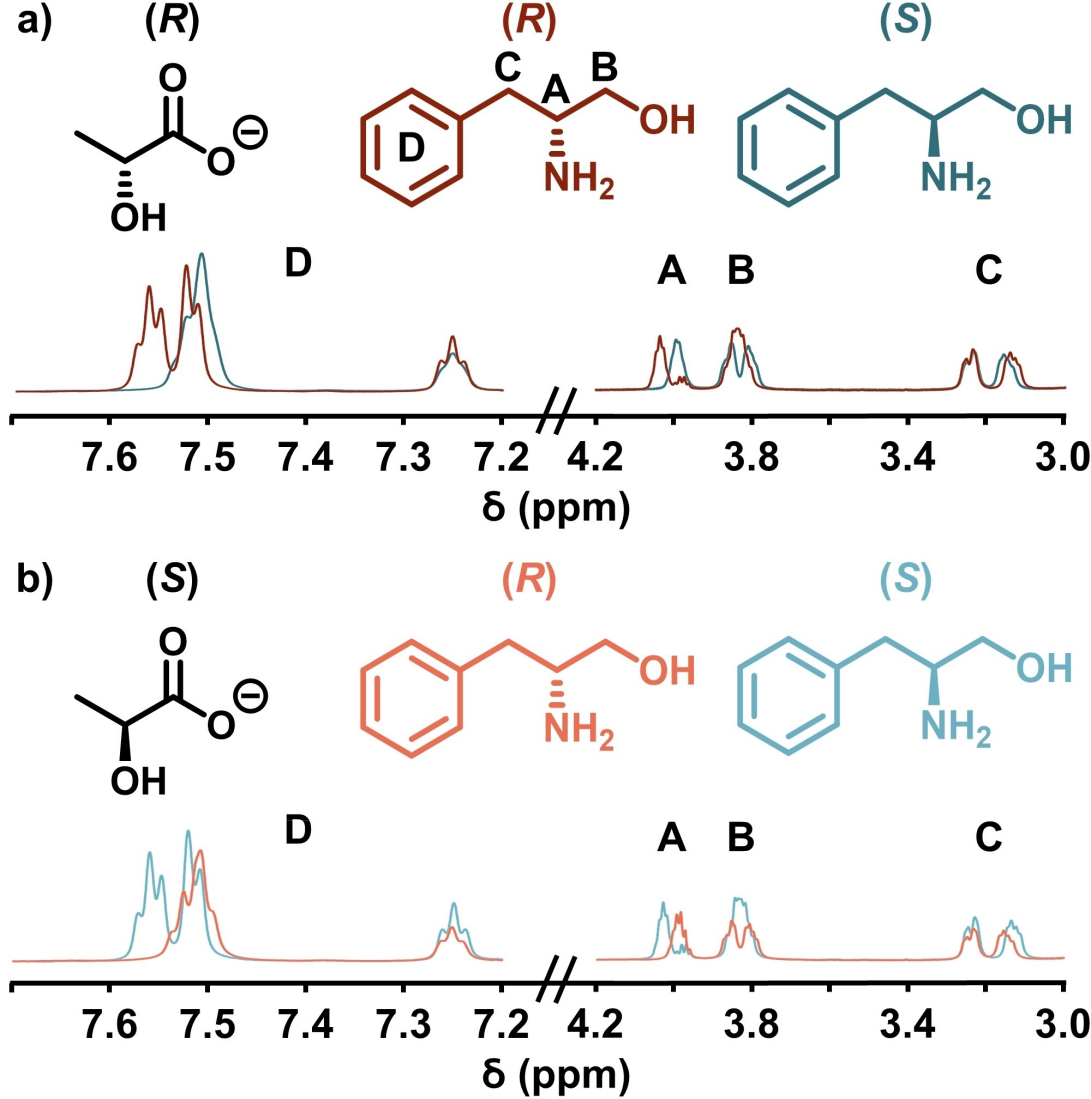
Partial ^1^H NMR spectra of enantiopure (*R*)‐ (red/orange) and (*S*)‐phenylalaninol (blue/light blue) guests with enantiopure a) {Mo_132_((R)‐lactate)_30_} and b) {Mo_132_((S)‐lactate)_30_} hosts at 278 K. The peaks related to the A, B, C, and D protons of the guest species have been highlighted. The two host enantiomers used are differentiated by the different shades of the two guest species.

The separation of pure enantiomers of phenylalaninol by (*R*)‐ or (*S*)‐lactate‐containing {Mo_132_} host structures was first investigated. The phenylalaninol peak positions shift downfield between *δ*=0.05 (CH(D)) and 1 ppm (CH(A)), from those positions exhibited by the guests in D_2_O only (Figure 2). Crucially, no effect on the free or bound lactate ligands was observed.

Next the ability of the chiral hosts to effect chiral resolution was investigated. Peaks relating to the proton of the chiral centre of each guest isomer showed magnetic inequivalence when the {Mo_132_((*R*)‐lactate)_30_} host was used: CH(D) ΔΔ*δ* −0.02 ppm, CH(A) ΔΔ*δ* −0.05 ppm, and CH_2_(B) ΔΔ*δ* −0.03 ppm, with the (*R*)‐phenylalaninol isomer more downfield shifted (Figure 2a). Importantly, when the {Mo_132_((*S*)‐lactate)_30_} host is used, these peak positions show opposite behaviour: CH(D) ΔΔ*δ*+0.02 ppm, CH(A) ΔΔ*δ*+0.04 ppm, and CH_2_(B) ΔΔ*δ*+0.01 ppm, respectively (Figure 2b). These results indicate that the separation of peaks is dependent upon the host isomer, with each host type having effectively opposite effects on the peak positions of the guest species, allowing for recognition of guest isomers. Additionally, guest phenyl peak positions, D, exhibit opposite multiplet patterns with each host isomer, serving as a secondary spectroscopic example of host‐guest isomer recognition.

The largest induction effect occurs for the protons at the chiral centre of the guest (CH(A)), indicating that this position is most likely closest to the interaction site with the {Mo_132_} host, while the phenyl protons show the lowest overall shift, indicating that this group is furthest from the interaction site. This information may mean that the guest is orientated with the protonated amine groups closest to the {Mo_2_} pore and the phenyl group furthest from the pore.

For fast‐exchange systems in NMR spectroscopy, lowering sample temperature generally leads to an increase in observed separations due to the reduced rate of exchange between the two possible guest environments. The above measurements were recorded at 300 K and therefore they were repeated at lower temperatures (278 K) to investigate this effect. The peak associated with proton CH(A)∼*δ* 4.1 ppm, located at the chiral centre of the guest molecule, showed distinct separation with both host enantiomers when the sample temperature was lowered. The separation, as taken between the two highest peaks, was ΔΔ*δ* −0.060 ppm for the {Mo_132_((*R*)‐lactate)_30_} host and ΔΔ*δ* +0.058 ppm for the {Mo_132_((*S*)‐lactate)_30_} host. Although small, these values are comparable to those observed for amino alcohols in D_2_O with alternative CSR hosts, such as resorcinarenes (0.018–0.347 ppm).[^34^, ^36^]

**Figure 3:**
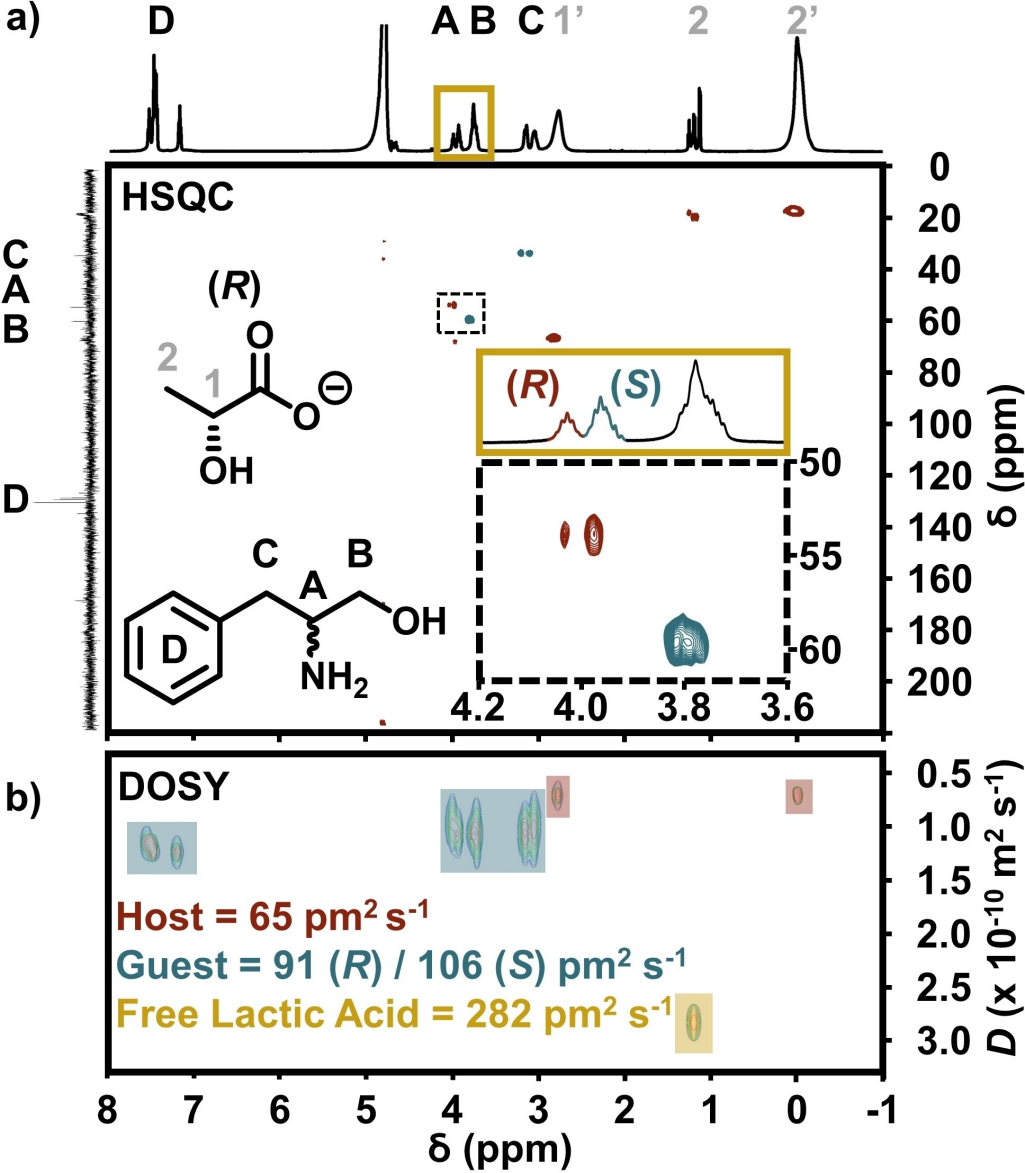
a) HSQC NMR spectrum of (*R*/*S*)‐phenylalaninol with the {Mo_132_((*R*)‐(lactate)_30_} host at 278 K. Separation of the proton resonance of the chiral centre (CH(A)), related to the *R* and *S* guest species, is highlighted in both the ^1^H NMR and HSQC spectra, by the yellow inset and dashed box insets, respectively. The assignment of the CH(A) proton resonance was later confirmed by using scalemic mixtures. b) DOSY NMR under the same conditions, highlighting the differences in diffusion coefficient of the host ligands (red boxes), guest species (blue boxes), and the free host ligand species (yellow box).

In order to determine the feasibility of the {Mo_132_(lactate)_30_} hosts as effective chiral shift reagents, the non‐equivalence of racemic guest peaks should be achieved. Addition of (*R*/*S*)‐phenylalaninol to either enantiopure {Mo_132_((*R*)‐lactate)_30_} or {Mo_132_((*S*)‐lactate)_30_} results in an expected similar overall shifting of peak positions, as observed for the addition of the enantiomerically pure guests. Comparison of the (*R*/*S*)‐phenylalaninol spectra with the enantiopure guest species and {Mo_132_((*R*)‐lactate)_30_} host highlights that the racemic guest mixture displays peaks which appear to be superimpositions of the two peak profiles exhibited for the enantiopure guests (Figure S22a). The phenyl peak region (CH(D)∼*δ* 7.6 ppm) of the guest exhibits a peak profile which is different from those of the enantiopure guest species, appearing to contain two separate overlapping peaks, with minimal separation. The guest peaks relating to the two CH_2_ groups (B∼*δ* 3.8 ppm and C∼*δ* 3.2 ppm) show no significant differences to those observed for the enantiopure guests with {Mo_132_((*R*)‐lactate)_30_}. The peak related to the proton at the chiral centre (CH(A)∼*δ* 4.1 ppm) appears to contain two distinct peaks, which are related to the two isomer guest forms undergoing chiral recognition by the host. For {Mo_132_((*S*)‐lactate)_30_} with the same (*R*/*S*)‐phenylalaninol guest mixture, broadly similar peak positions and profiles were observed as described for the {Mo_132_((*R*)‐lactate)_30_} host (Figure S22b). For each host species, there is no notable effect on the peak profiles of either the free or coordinated lactate ligands upon addition of the racemic guest species. The peak assignments and separations described were reflected in the HSQC spectrum at 278 K (Figure 3a). The resonance separation effect described in the ^1^H NMR spectra and consequent experiments were also reflected in DOSY NMR data (Figure 3b). Due to their encapsulation within the {Mo_132_} framework, the diffusion coefficient of the coordinated lactate ligands should effectively represent the diffusion coefficient of the POM framework itself. When the phenylalaninol guests are present, the lactate ligands possess a diffusion coefficient of approximately 65 pm^2^ s^−1^ for the (*R*)‐lactate host and 63 pm^2^ s^−1^ for the (*S*)‐Lactate host. The free (*R*)‐ and (*S*)‐lactic acid possess diffusion coefficients of 282 and 275 pm^2^ s^−1^, respectively. Upon addition of (*R*/*S*)‐phenylalaninol to a solution of {Mo_132_((*R*)‐lactate)_30_}, the (*R*)‐guest molecule displays a diffusion coefficient of 91 pm^2^ s^−1^, whereas (*S*)‐phenylalaninol has a diffusion coefficient of 106 pm^2^ s^−1^. Similarly, addition of the guest to {Mo_132_(*S*)‐lactate)_30_} gives diffusion coefficients of 92 and 88 pm^2^ s^−1^ for the *R* and *S* guests, respectively. These results demonstrate significant guest interaction as well as a clear preference for homochiral recognition. That the (*S*)‐lactate host exhibits lower overall diffusion coefficients for both guests compared to the (*R*)‐lactate host, coupled with the greater downfield shift observed for the guest species with {Mo_132_((*S*)‐lactate)_30_} in the ^1^H NMR spectra, indicates that the strength of interaction of the phenylalaninol guests with {Mo_132_(*S*)‐lactate)_30_} is stronger than that of the same guests with the (*R*)‐lactate‐containing structure. However, the overall separating ability of each host structure is similar, although reversible in nature.

We were able to determine an approximate association constant (*K*_a_) using a single‐spectrum DOSY NMR method for the phenylalaninol guest species. This determination relied on an assumption of a 1 : 1 binding model between the guest and each of the host pore sites. Although analysis was performed to determine the binding ratio experimentally, a reliable figure could not be obtained due to a loss of spectral resolution above a 1 : 1 binding ratio. We expect the binding strength will be affected by allosteric effects of adjacent pore sites due to the concurrent sharing of individual ligands between two sites. For our calculation (see the Supporting Information for more details), the diffusion coefficient of the free guest species (*D*_f_) was taken as the value of the free lactic acid ligands, while the value corresponding to the {Mo_132_} host was taken as the value of the coordinated lactate ligands (*D*_b_). The observed diffusion coefficient of the guest (*D*_o_), the total guest concentration (*G*_TOT_), and the total host concentration (*H*_TOT_) are then used to determine the dissociation constant (*K*_d_) and related association constant (*K*_a_) according to Equation 1:

(1) $$K_{d}=\frac{1}{K_{a}}=H_{\mathrm{TOT}}\left(\frac{D_{b}-D_{o}}{D_{o}-D_{f}}\right)+G_{\mathrm{TOT}}\left(\frac{D_{o}-D_{b}}{D_{b}-D_{f}}\right)$$

The association constants of the *R* and *S* guests with the {Mo_132_((*R*)‐lactate)_30_} host were 350(±5) and 190(±2) M^−1^, respectively. With the {Mo_132_((*S*)‐lactate)_30_} hosts, these values were effectively reversed, with 270(±2) and 390(±11) M^−1^ for the (*R*) and (*S*)‐phenylalaninol guests, respectively (Table 1).

**Table 1:** ^1^H NMR shift separation of racemic guests, (*R*/*S*)‐phenylalaninol and (*R*/*S*)‐2‐amino‐1‐phenylethanol, with enantiopure {Mo_132_} host structures with different coordinated ligands. Association constants for the *R* (red) and *S* (blue) guests derived from DOSY NMR data are also given. All values were obtained at 278 K.

	Peak separation, ΔΔ*δ* (ppm)		Association constant, *K*_a_ [M^−1^]	
	Host isomer		Host isomer	
*R*/*S* guest	*R*	*S*	*R*	*S*
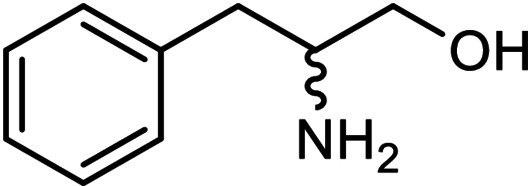	−0.060	+0.058	350±5 190±2	270±2 390±11
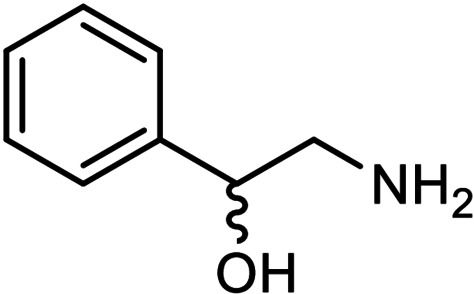	−0.021	+0.022	110±3 110±3	55±9 60±9

### Chiral recognition of other chiral amino alcohols using {Mo_132_(lactate)_30_} hosts

After confirming the recognition of phenylalaninol isomers we next turned our attention to a range of different amino alcohol guests. Using 2‐amino‐1‐phenylethanol, smaller ΔΔ*δ* values were observed suggesting a weaker interaction at the {Mo_132_} pore (Table 1). This can be rationalised by the differences in guest structure as the key amine group in phenylalaninol is bound to the chiral centre while for 2‐amino‐1‐phenylethanol the amine is α to the chiral centre. Additionally, the association constants for the interaction of this guest with the host species has been derived and is given in Table 1. For other amino alcohol guests (leucinol, 2‐aminohexan‐1‐ol, and 3‐isopropylaminopropane‐1,2‐diol) no significant separation of the guest peaks was observed. These guests lack bulky substituents such as the phenyl rings found in the separated phenylalaninol and 2‐amino‐1‐phenylethanol, suggesting that this may be another important factor in promoting the separation behaviour.

### Scalemic phenylalaninol mixtures for determination of enantiomeric excess

**Figure 4:**
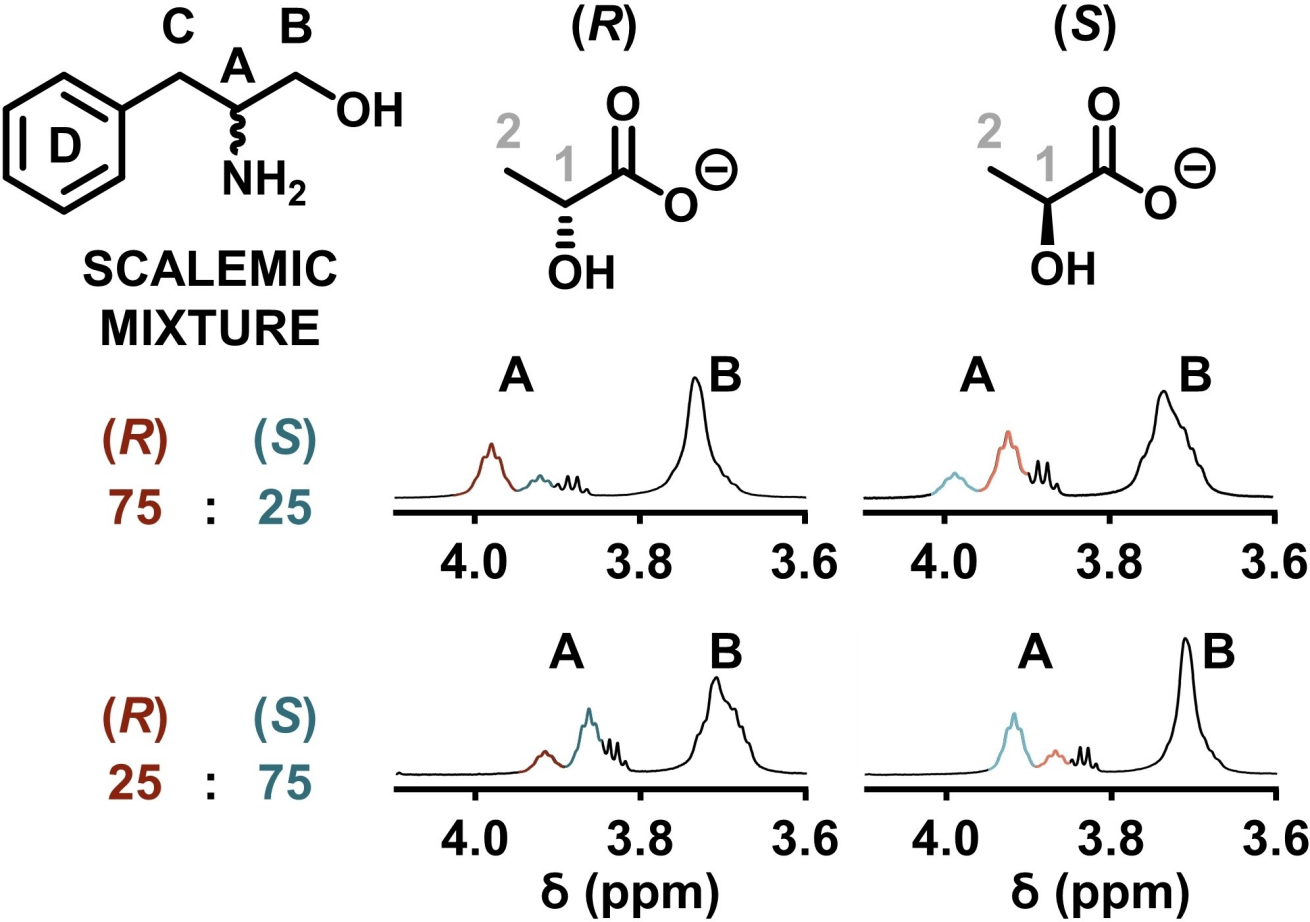
^1^H NMR spectra of scalemic mixtures of (*R*) and (*S*)‐phenylalaninol with enantiopure {Mo_132_(lactate)_30_} host structures at 278 K. The CH(A) proton was assigned by observing the effects of changing the ratio of the guest mixture components on resonance profiles. In addition, shift reversibility as a result of changing host enantiomers was also observed.

To confirm from which enantiomer each separated CH(A) peak of the racemic amino alcohol guests arises, nonracemic (scalemic) mixtures of the guest were added to the solutions. In a similar manner to those experiments with the racemic mixtures, the total number of guest equivalents added were maintained at twelve times the concentration of the {Mo_132_} host, with ratios of *R* to *S* guests of 3 : 1, and 1 : 3, for two separate reactions, applied. Low temperature (278 K) ^1^H NMR spectroscopy was applied to probe the resulting solutions (Figure 4).

Firstly, for the {Mo_132_((*R*)‐lactate)_30_} host, when the ratio of *R* to *S* guest added was 3 : 1, the CH(A) peak related to the proton at the stereogenic centre (∼*δ* 4.1 ppm), displays the previously observed two peaks, with the more downfield shifted peak more intense than the other, attributing these to the *R* and *S* guest, respectively (Figure 4a). Therefore, the CH(A) signals in both the racemic and scalemic samples at 278 K with the {Mo_132_((*R*)‐lactate)_30_} host, the more downfield shifted peak is attributed to the (*R*)‐phenylalaninol guest, indicating that the extent of shift separation is (−ΔΔ*δ* 0.06 ppm). This assessment can be confirmed by comparing this spectrum to that of the *R : S* guest in a 1 : 3 ratio, with the {Mo_132_((*R*)‐lactate)_30_} host. Here, the more intense peak is the more upfield shifted peak which is derived from (*S*)‐phenylalaninol.

Comparison of the above (*R*)‐ and (*S*)‐phenylalaninol scalemic mixture samples with the analogous (*S*)‐lactate‐containing {Mo_132_} host, was also performed (Figure 4b). For the *R* to *S* guest ratio of 3 : 1, the more intense peak is now the more upfield shifted peak, indicating that the extent of separation with the {Mo_132_((*S*)‐lactate)_30_} host is +ΔΔ*δ* 0.06 ppm, with the *S* guest more downfield shifted. Again, by comparison of these results with the 1 : 3 (*R*)‐ to (*S*)‐phenylalaninol mixture, the more intense peak here is the more downfield shifted resonance, confirming that this peak relates to that of the (*S*)‐phenylalaninol guest. These measurements confirm that the separation of chiral amino alcohol guest species is entirely dependent upon the configuration of the ligands present on the {Mo_132_} host structure.

A similar measurement was performed for the (*R*) and (*S*)‐2‐amino‐1‐phenylethanol guests, with the resulting spectra indicating that the most downfield shifted guest is the *R* isomer when the {Mo_132_((*R*)‐lactate)_30_} host is used, and the *S* guest is the most downfield shifted peak when the {Mo_132_((*S*)‐lactate)_30_} host is used (Figure S32).

We were able to perform crude integration of the two peaks observed, roughly determining the enantiomeric excess of guest added to each sample. For the {Mo_132_((*R*)‐lactate)_30_} host, with the (*R*)‐ to (*S*)‐phenylalaninol guest added in a 3 : 1 ratio, the resulting integral ratios were 3:0.95, while for the 1 : 3 ratio the integral ratio was 0.96 : 3. These results therefore highlight the ability of the {Mo_132_(lactate)_30_} system to determine approximate enantiomeric excesses of these amino alcohol species.

### Control reactions

**Figure 5:**
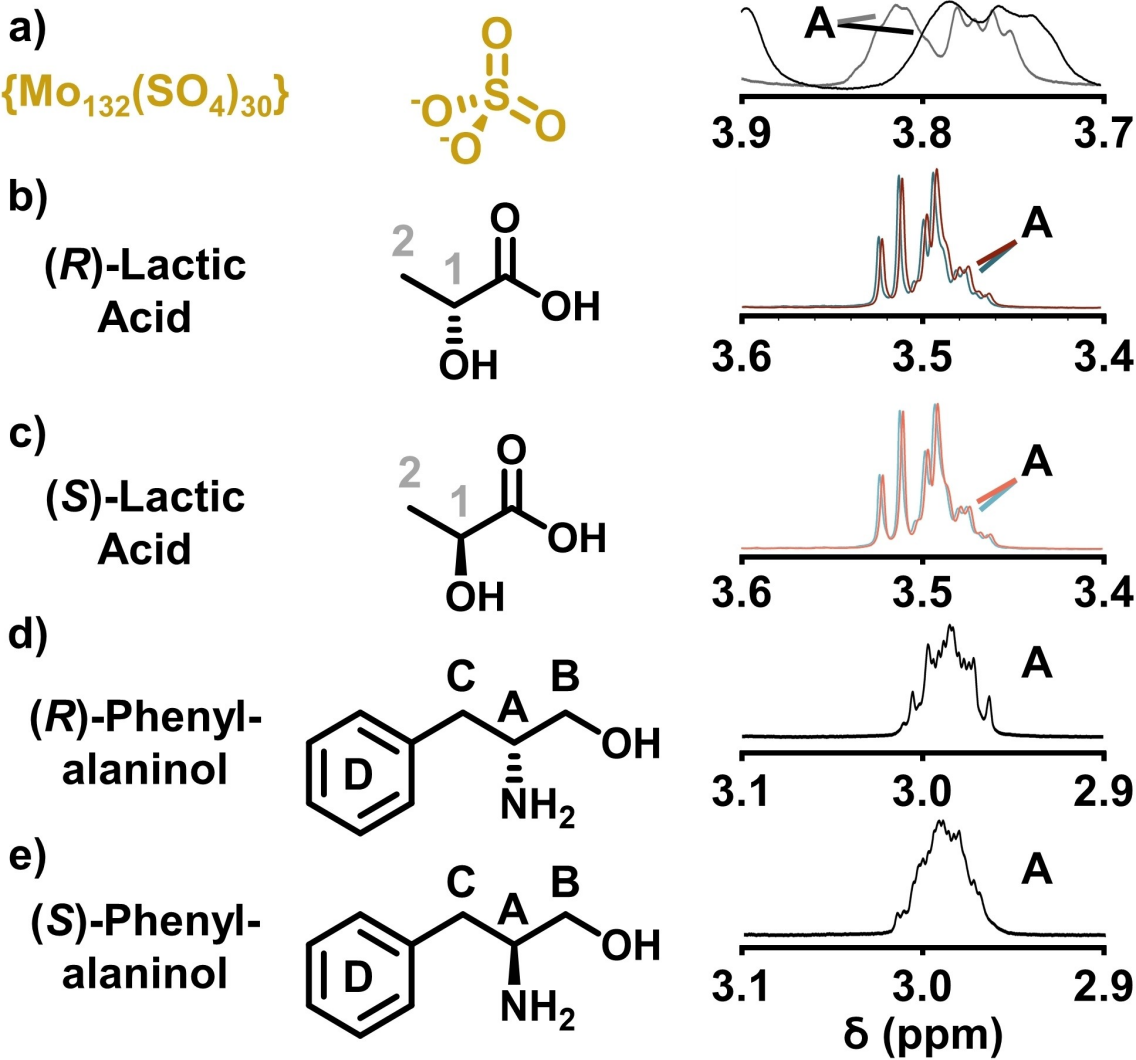
Selected ^1^H NMR spectra of the proton resonance at the guest chiral centre of a) {Mo_132_(SO_4_)_30_} and (*R*)‐ (black line), and (*S*)‐phenylalaninol (grey line), b) (*R*)‐lactic acid and (*R*)‐ (red line), and (*S*)‐phenylalaninol (blue line), c) (*S*)‐lactic acid and (*R*)‐ (red line), and (*S*)‐phenylalaninol (blue line), d) (*R*)‐phenylalaninol only, and e) (*S*)‐phenylalaninol only, control reactions.

After investigating the effect of racemic guest isomer addition to our {Mo_132_(lactate)_30_} system, several control reactions were performed. The first of which involved the separate addition of enantiopure phenylalaninol guest isomers to a solution of D_2_O containing the enantiomerically pure (*R*) or (*S*)‐lactic acid ligands only (Figure 5b, c). This was performed to determine the influence of the free lactic acid ligands, which are found in aqueous solutions of the dissolved {Mo_132_} structures, on the guest peak positions. The concentration of lactic acid was chosen to match that of the concentration present when the {Mo_132_} species was investigated (30×5 mM=150 mM). The phenylalaninol peak positions shifted downfield by CH(D) Δ*δ* 0.0/0.0 ppm, CH_2_(B) Δ*δ* +0.2/0.2 ppm, CH(A) Δ*δ* +0.5 ppm (slightly obscured by the CH_2_(B) resonances), and CH_2_(C) Δ*δ* +0.2/0.5 ppm, respectively, from those positions exhibited by the phenylalaninol guests in D_2_O only. A lack of enantiomer peak separation here indicated that the influence of the free lactic acid molecules is limited to non‐specific shifts exhibited by each guest isomer.

In another control experiment, the enantiopure phenylalaninol isomers were added separately to a solution containing {Mo_132_(SO_4_)_30_} in D_2_O only (Figure 5a). This was performed to determine the extent of shift effects provided by the {Mo_132_} framework itself, when non‐chiral hydrophilic ligands are coordinated. The peak positions shifted, from those discussed for the enantiopure phenylalaninol isomers in D_2_O only, by CH(D) Δ*δ* +0.15/0.07 ppm, CH_2_(B) Δ*δ* +0.4/0.5 ppm, CH(A) Δ*δ* +0.8 ppm, and CH_2_(C) Δ*δ* +0.45/0.75 ppm, respectively. All peaks of each isomer showed slight separation from one another of CH(D) ΔΔ*δ* +0.02/+0.01 ppm, CH_2_(B) ΔΔ*δ* +0.02/+0.02 ppm, CH(A) ΔΔ*δ* +0.03 ppm, and CH_2_(C) ΔΔ*δ* +0.02 ppm, with the (*S*)‐phenylalaninol isomer more downfield shifted for all peaks. This increased shift separation indicates that the {Mo_132_} host structure itself facilitates isomer separation, which may be caused by the formation of diastereoisomers either by interaction of guests with neighbouring pores or by guest facilitating stereoselective sulfate ligand hydrolysis to create an axially chiral pore. Unfortunately, an experimental basis to understand this phenomenon has not yet been achieved, however we are interested in understanding this behaviour as an ongoing endeavour. It should be noted that the overall shifting of the guest peaks is increased when either of the {Mo_132_(lactate)_30_} hosts are used, highlighting the increased interaction strength when the lactate ligands are present, which is subsequently manifested in the extent of peak separation.

## Conclusion

We have successfully isolated and characterised new chiral‐containing {Mo_132_} spherical‐type POM structures. In doing so, we have used extensive NMR methods to characterise the structures in the solution state. Following this, we used these newly functionalised species to enantioselectively recognise cationic amino alcohol guests in aqueous solvent, performing the role of a chiral shift reagent. Although solid‐state characterisation of the host‐guest interaction could not be achieved, we hypothesise that the electrostatic interaction between the cationic ammonium group of the guest and the electronegative {Mo_132_} host, coupled with hydrogen bond interactions between host‐bound chiral lactate ligands and guest isomers, facilitates the formation of diastereomeric complexes. The resulting separation process is comparable to previously reported methods that exclusively used organic species and were primarily limited to nonaqueous solvents, representing a new application of the POM species. The work completed here focused on the role of the {Mo_2_}‐formed pore as the site for interaction with chiral guests and therefore investigation of the internal cavity as a second site for chiral discrimination is a viable potential extension of this methodology, with a focus on the recognition of guests with more diverse functionalities.

## Author Contributions

The idea for the work was conceived by L.C., the synthesis was done by Z.L.S., the main hypothesis development and work plan and NMR measurements by R.W.P. with help from N.W., N.L.B. and Y.M.A. Structural analysis was performed by Z.L.S. and D.L. D.L. and N.L.B. led the clusters team a with help from L.C. The manuscript was written by R.W.P. with input from all the authors.

## Experimental Section

All experimental procedures, data and equipment parameters can be found in the Supporting Information. Deposition Number 1898694 contains the supplementary crystallographic data for this paper. These data are provided free of charge by the joint Cambridge Crystallographic Data Centre and Fachinformationszentrum Karlsruhe Access Structures service.

## Conflict of interest

The authors declare no conflict of interest.

## Supporting information

As a service to our authors and readers, this journal provides supporting information supplied by the authors. Such materials are peer reviewed and may be re‐organized for online delivery, but are not copy‐edited or typeset. Technical support issues arising from supporting information (other than missing files) should be addressed to the authors.

Supporting Information
