# Supplementary material for: Enantioselective Recognition of Racemic Amino Alcohols in Aqueous Solution by Chiral Metal‐Oxide Keplerate {Mo132} Cluster Capsules
Source: Chemistry. 2021 Jul 30;27(48):12327–34. doi: 10.1002/chem.202100899 (PMC8457076; doi:10.1002/chem.202100899)
Supplement: Supplementary file 1 — Supporting Information [file CHEM-27-12327-s001.pdf]

Chemistry–A European Journal

Supporting Information

Enantioselective Recognition of Racemic Amino Alcohols in Aqueous Solution by Chiral Metal-Oxide Keplerate {Mo_{132}} Cluster Capsules

Robert W. Pow, Zoë L. Sinclair, Nicola L. Bell, Nancy Watfa, Yousef M. Abul-Haija, De-Liang Long, and Leroy Cronin*

Supplementary Information

Enantioselective Recognition of Racemic Amino Alcohols in Aqueous Solution by Chiral Metal-Oxide Keplerate {Mo_{132}}-cluster Capsules

Contents

- Materials 2
- Single Crystal X-Ray Crystallography 2
- Infrared Spectroscopy 2
- pH Measurements 2
- Elemental Analysis 2
- Nuclear Magnetic Resonance Spectroscopy 2
- Chiral Guest with {Mo_{132}(Lactate)$_{30}$} NMR Preparation Procedure 2

Synthesis of the Capsules 3

- Synthesis of {Mo_{132}(OAc)$_{30}$} for Ligand-Exchange Syntheses and ^{1}H NMR Ligand-Exchange Studies 3
- Synthesis of {Mo_{132}(SO_4)$_{30}$} from {Mo_{132}(OAc)$_{30}$} for use in Ligand-Exchange Syntheses 3
- Synthesis of {Mo_{132}(Lactate)$_{30}$} Structures 3
- Structural Formulae of {Mo_{132}(Lactate)$_{30}$} 3

Solid-State Structure Characterisation 3

- Circular Dichroism 4
- Crystallographic Data 4
- Thermogravimetric Analysis 4
- Infrared Spectroscopy 6

Solution-State Structure Characterisation 7

- Nuclear Magnetic Resonance Spectroscopy 7

Chiral Recognition by {Mo_{132}(Lactate)$_{30}$} Host Pores via ^{1}H NMR 9

- Amino Alcohol Guest Selection 9
- Scheme of Shift Separation Assignment 9
- Control Reactions 10
- Separation of Enantiopure Amino Alcohol Guests with Enantiopure {Mo_{132}(Lactate)$_{30}$} Host Structures 13
- Separation of Racemic Amino Alcohol Guests with Enantiopure {Mo_{132}(Lactate)$_{30}$} Host Structures 18
- Separation of Scalemic Amino Alcohol Guests with Enantiopure {Mo_{132}(Lactate)$_{30}$} Host Structures 27

Determination of Association Constant of Amino Alcohols with {Mo_{132}(Lactate)$_{30}$} 29

Materials and Instrumentation

Materials

All reagents and solvents were purchased from Sigma-Aldrich Chemical Company Ltd., Alfa Aesar, and Tokyo Chemical Industries. Materials were used without further purification.

Single Crystal X-Ray Crystallography

A small amount of solution containing crystals was isolated by pipetting onto a glass slide. Excess solvent was removed and Fomblin oil was quickly added to coat the crystals. The Fomblin oil serves a dual role of protecting the crystals during mounting and cooling, and as an adhesive during crystal mounting. A suitable single crystal was selected and mounted onto a rubber loop. X-ray diffraction intensity data were collected on a Bruker Apex 2 CCD diffractometer (λ (MoK_α) = 0.7107 Å) equipped with a microfocus X-ray source (50 kV, 30 w). Data collection and reduction were performed using the Apex3 or CrysAlisPro software package, and structure solution and refinement was carried out by SHELXS-2014 and SHELXL-2014 using WinGX. Corrections for incident and diffracted beam absorption effects were applied using empirical absorption correction. All non-hydrogen atoms (including those disordered) were anisotropically refined. All structures were collected and solved by Zoë Sinclair and final refinements by Deliang Long. The X-ray crystallographic data reported in this article have been deposited at the Crystallographic Data Centres. For {Mo_{132}((*S*)-Lactate)$_{30}$}, the data can be obtained free of charge from the Cambridge Crystallographic Data Centre via www.ccdc.cam.ac.uk/data_request/cif under deposition number CCDC-1898694.

Infrared Spectroscopy

All samples were collected in transmission mode using a JASCO FT-IR-410 spectrometer.

pH Measurements

Measurements were taken on a Hanna Instruments HI-2210-02 Bench Top pH Meter with pH electrode (HI 1131B) and temperature probe (HI 7662).

Elemental Analysis

Element analyses for Mo and S were performed on a Leeman inductively-coupled plasma (ICP) spectrometer. Carbon, nitrogen and hydrogen content were determined by the microanalysis services within the School of Chemistry, University of Glasgow, using an EA 1110 CHNS, CE-440 Elemental Analyzer.

Nuclear Magnetic Resonance Spectroscopy

^{1}H, ^{13}C and associated NMR spectra were recorded on a Bruker Ascend Aeon 600 MHz NMR spectrometer. Samples were analysed in Bruker NMR tubes (5 mm diameter, 7” length). Unless otherwise stated, 1.7 x 10^{-3} M solutions of the {Mo_{132}}-based structures were prepared by dissolving the relevant sample in D_2O. Where required, a methanesulfonic acid or maleic acid reference solution of concentration 3.4 x 10^{-2} M (i.e. 20x the concentration of the {Mo_{132}} solution) was prepared and transferred to an external axial reference tube.

Chiral Guest with {Mo_{132}(Lactate)$_{30}$} NMR Preparation Procedure

{Mo_{132}(Lactate)$_{30}$} (33 mg, 1.1 x 10^{-3} mmol), prepared as below, was dissolved in D_2O (0.4 mL) and added to an NMR tube (5 mm diameter, 7” length). Guest solutions were added in 100 µL increments, with shaking, with concentrations as a factor of a specific number of equivalents in comparison to the concentration of the {Mo_{132}(Lactate)$_{30}$} species. After 1 minute the samples were ready to be analysed via NMR.

Synthesis of the Capsules

Synthesis of $\{Mo_{132}(OAc)_{30}\}$ for Ligand-Exchange Syntheses and ^1H NMR Ligand-Exchange Studies

Ammonium heptamolybdate tetrahydrate [$(NH_4)_6Mo_7O_{24}.4H_2O$] (5.6 g, 4.5 mmol) and ammonium acetate [CH_3COONH_4] (12.5 g, 162 mmol) were added to a wide-necked 500 mL conical flask containing deionised water (250 mL) and a magnetic stirrer bar. Acetic acid [CH_3COOH] (50%) was added, under stirring, until the solution reached pH 4 (83 mL). Finally, hydrazine sulphate [$N_2H_4.H_2SO_4$] (0.8 g, 6.1 mmol) was added and the solution was stirred at room temperature for 10 minutes. The resulting dark brown solution was transferred to a temperature-controlled environment (18°C) and left uncovered to allow solvent evaporation to occur. After 2 days, dark brown hexagonal crystals had formed which were suitable for single-crystal X-ray diffraction data collection – used here to confirm the expected unit cell of the $\{Mo_{132}\}$ structure. The crystals were collected and washed with a minimum amount of ethanol followed by diethyl ether, then dried in air. Yield: 4.04 g (60.6%, based on Mo).

Synthesis of $\{Mo_{132}(SO_4)_{30}\}$ from $\{Mo_{132}(OAc)_{30}\}$ for use in Ligand-Exchange Syntheses

$\{Mo_{132}(OAc)_{30}\}$ (4.0 g, 0.14 mmol), prepared as above, and ammonium sulphate [$(NH_4)_2SO_4$] (16.0 g, 121.1 mmol) were added to a wide-necked 500 mL conical flask containing deionised water (320 mL) and a magnetic stirrer bar. Addition of hydrochloric acid [HCl] (16%), under stirring, produced a pH 1 solution (16 mL), which was then stirred at 90°C for 4 hours. After stirring, the dark brown solution was filtered whilst hot, and the filtrate was transferred to a temperature-controlled environment (18°C) for storage and left uncovered to allow for solvent evaporation to occur. After 4 days, dark brown hexagonal crystals had formed which were suitable for single-crystal X-ray diffraction data collection – used here to confirm the expected unit cell of the $\{Mo_{132}\}$ structure. The crystals were collected and washed with a minimum amount of ethanol followed by diethyl ether, then dried in air. Yield: 1.03 g (25.8%).

Synthesis of $\{Mo_{132}(Lactate)_{30}\}$ Structures

Syntheses and solid state characterisation of the $\{Mo_{132}(Lactate)_{30}\}$ structures were performed by Zoë Sinclair. $\{Mo_{132}(SO_4)_{30}\}$ (500 mg, 1.8×10^{-2} mmol), prepared as above, was added to a wide-necked 50 mL conical flask containing deionised water (20 mL) and a magnetic stirrer bar. The desired ligand for use in each reaction was added in the following amounts: (*R*)-lactic acid – 184 µL, 4.8 mmol (250 eq.), (*S*)-lactic acid – 184 µL, 4.8 mmol (250 eq.). The number of equivalents in brackets is the approximate number of equivalents of ligand added in comparison to the number of moles of $\{Mo_{132}(SO_4)_{30}\}$. The pH of the solutions were adjusted to pH 2.8 by addition of ammonium hydroxide (2M). After stirring at 50°C for 3 days, ammonium chloride (NH_4Cl) – 230 mg, 4.3 mmol, was added and the solutions were stirred for a further 10 mins. The solutions were then transferred to a temperature-controlled environment (18°C) for storage in their uncovered containers, to allow solvent evaporation to occur. Dark brown, hexagonal crystals which were suitable for single-crystal X-ray analysis appeared after a period of 5 days. The crystals were washed with a minimum amount of ethanol followed by diethyl ether, then dried in air. Yield = 70-90 mg (30-40%).

Structural Formulae of $\{Mo_{132}(Lactate)_{30}\}$

$\{Mo_{132}((R)\text{-Lactate})_{30}\}$: $(NH_4)_{46}[Mo_{132}O_{372}(H_2O)_{72}(C_3H_5O_3)_{30}](SO_4)_2(H_2O)_{250}$

$\{Mo_{132}((S)\text{-Lactate})_{30}\}$: $(NH_4)_{49}[Mo_{132}O_{372}(H_2O)_{72}(C_3H_5O_3)_{30}](C_3H_5O_3)_5(SO_4)(H_2O)_{220}$

Solid-State Structure Characterisation

Circular Dichroism

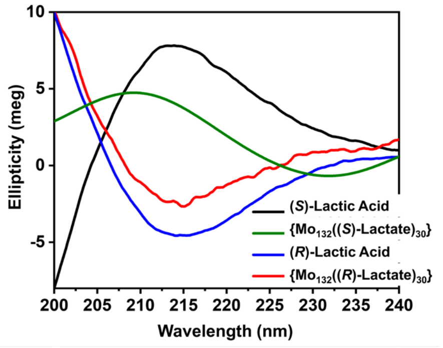

Figure 1: *CD spectra of (R)-Lactic acid (blue), (S)-Lactic acid (black), {Mo_{132}((R)-Lactate)$_{30}$} (red), and {Mo_{132}((S)-Lactate)$_{30}$} (green).*

Crystallographic Data

Identification Code	{Mo_{132}((*S*)-Lactate)$_{30}$} (CCDC-1898694)	
Empirical Formula	C_{105} H_{955} Mo_{132} N_{49} O_{773} S_2	
Formula Weight	27974.26	
Temperature	150(2) K	
Wavelength	0.71073 Å	
Crystal System	Trigonal	
Space Group	*R-3*	
Unit Cell Dimensions	a = 32.669(3) Å	α = 90°
	b = 32.669(3) Å	β = 90°
	c = 73.347(4) Å	γ = 120°
Volume	67793(11) Å^3	
Z	3	
Density (calculated)	2.056 Mg/m^3	
Absorption Coefficient	1.878 mm^{-1}	
F(000)	41016	
Crystal Size	0.100 x 0.100 x 0.100 mm^3	
Theta Range for Data Collection	1.925 to 25.999°	
Index Ranges	-40<=h<=40	40<=k<=40	-90<=l<=90
Reflections Collected	499094	
Independent Reflections	29610 [R(int) = 0.0401]	
Completeness to Theta	25.242° (99.9 %)	
Absorption Correction	Empirical	
Max./Min. Transmission	0.746 / 0.645	
Refinement Method	Full-Matrix Least-Squares on F2	
Data / Restraints / Parameters	29610	134	1444
Goodness-of-fit on F^2	1.190	
Final R Indices [I>2sigma(I)]	R1 = 0.0521	wR2 = 0.1365
R Indices (All Data)	R1 = 0.0608	wR2 = 0.1537
Extinction Coefficient	N/A	
Largest Diff. Peak and Hole	2.34	1.15 e.Å^{-3}

Thermogravimetric Analysis

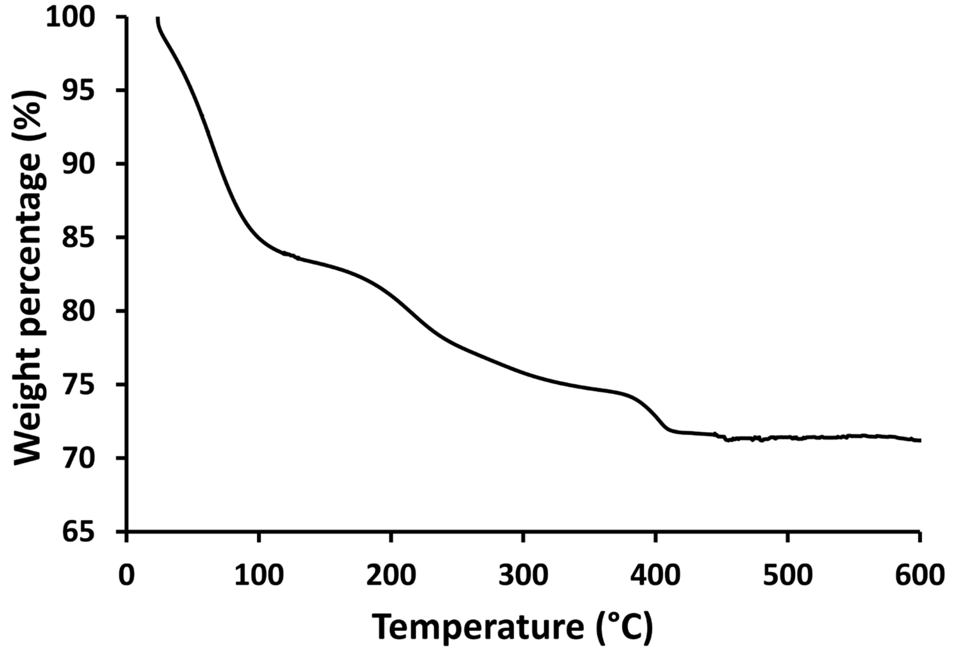

Figure 2: *Thermogravimetric analysis of {Mo_{132}((R)-Lactate)$_{30}$}.*

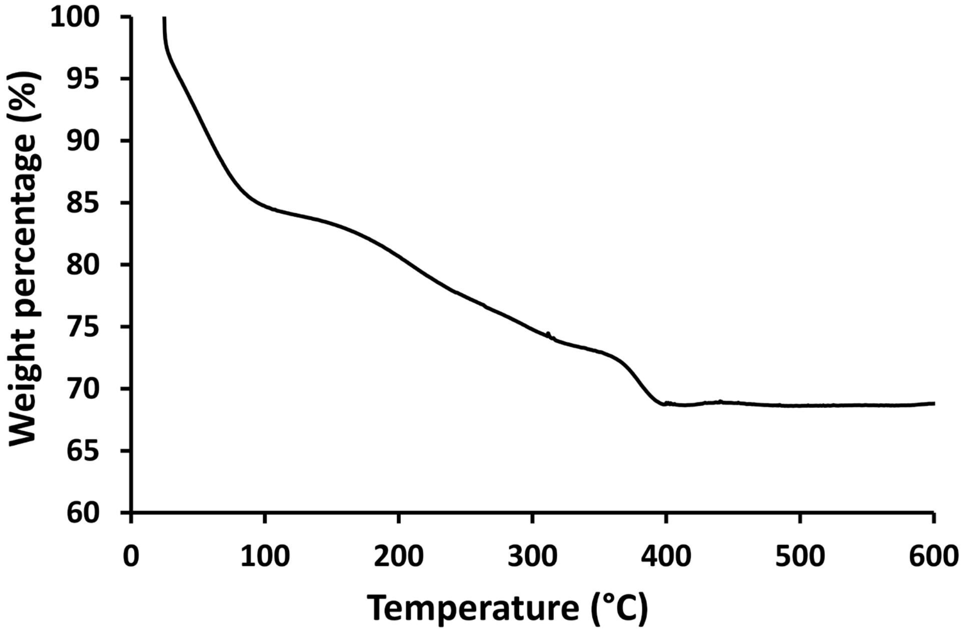

Figure 3: *Thermogravimetric analysis of {Mo_{132}((S)-Lactate)$_{30}$}.*

Infrared Spectroscopy

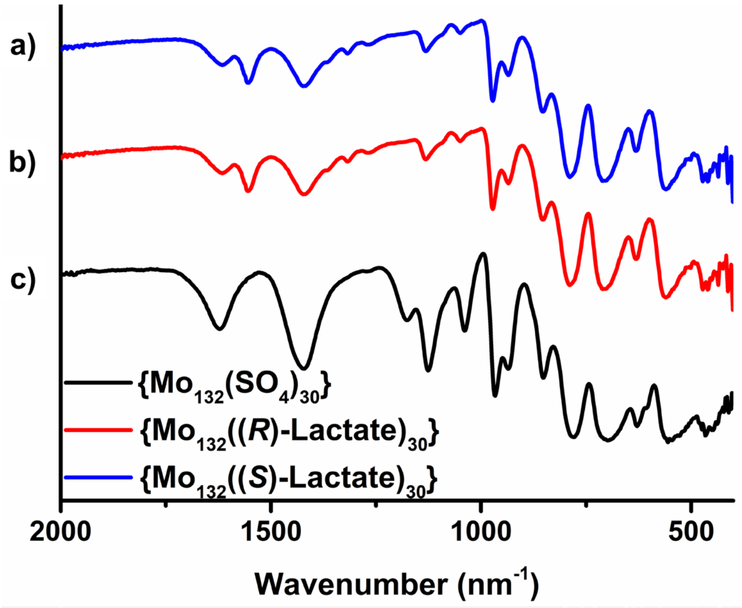

Figure 4: *IR spectra of selected structures: a) {Mo_{132}((R)-Lactate)$_{30}$}, b) {Mo_{132}((S)-Lactate)$_{30}$}, c) {$Mo_{132}(SO_4)_{30}$}.*

Solution-State Structure Characterisation

Nuclear Magnetic Resonance Spectroscopy

{Mo_{132}((R)-Lactate)$_{30}$} and (R)-Lactic Acid

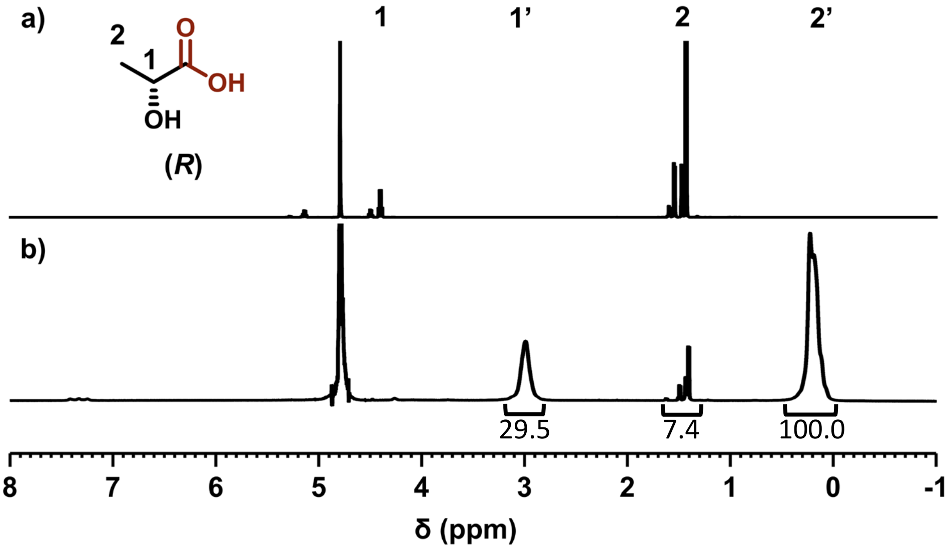

Figure 5: *^{1}H NMR of a) pure (R)-lactic acid, and b) {Mo_{132}((R)-Lactate)$_{30}$}. The shifted and broadened peaks related to the encapsulated ligands are highlighted as 1' and 2' for b).*

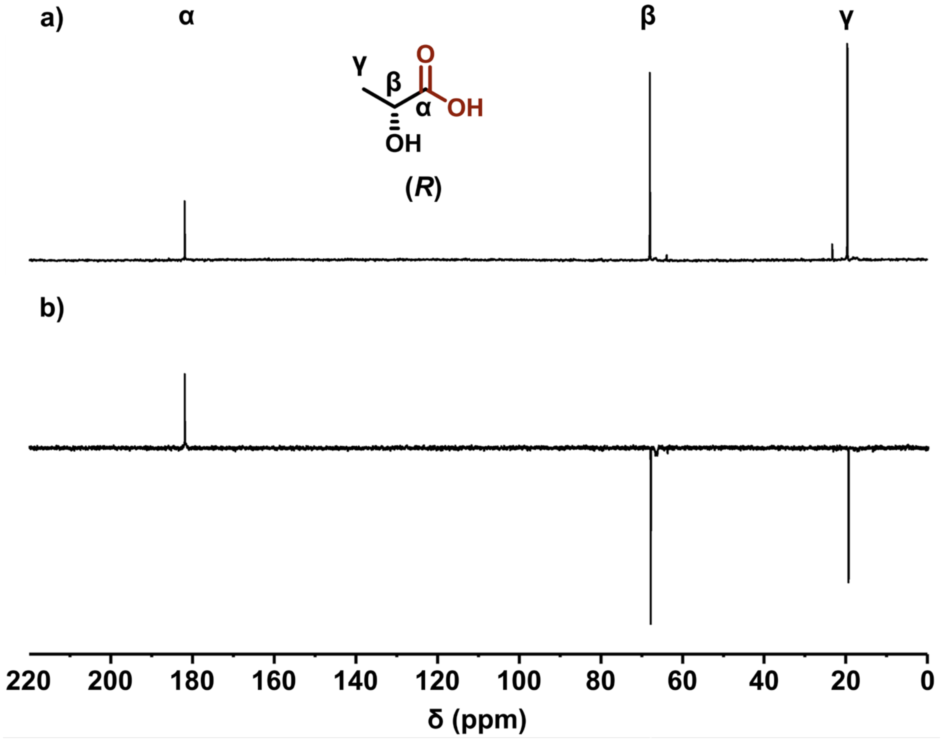

Figure 6: *^{13}C NMR of {Mo_{132}((R)-Lactate)$_{30}$}, and b) DEPTQ NMR of {Mo_{132}((R)-Lactate)$_{30}$} with C_q and CH_2 resonances pointing upwards, and CH and CH_3 resonances pointing downwards. The shifted and broadening effects observed in ^{1}H NMR spectra are not observed here due to the small differences between the two resonances. Peaks have been relabelled to differentiate between peaks in ^{1}H and ^{13}C spectra.*

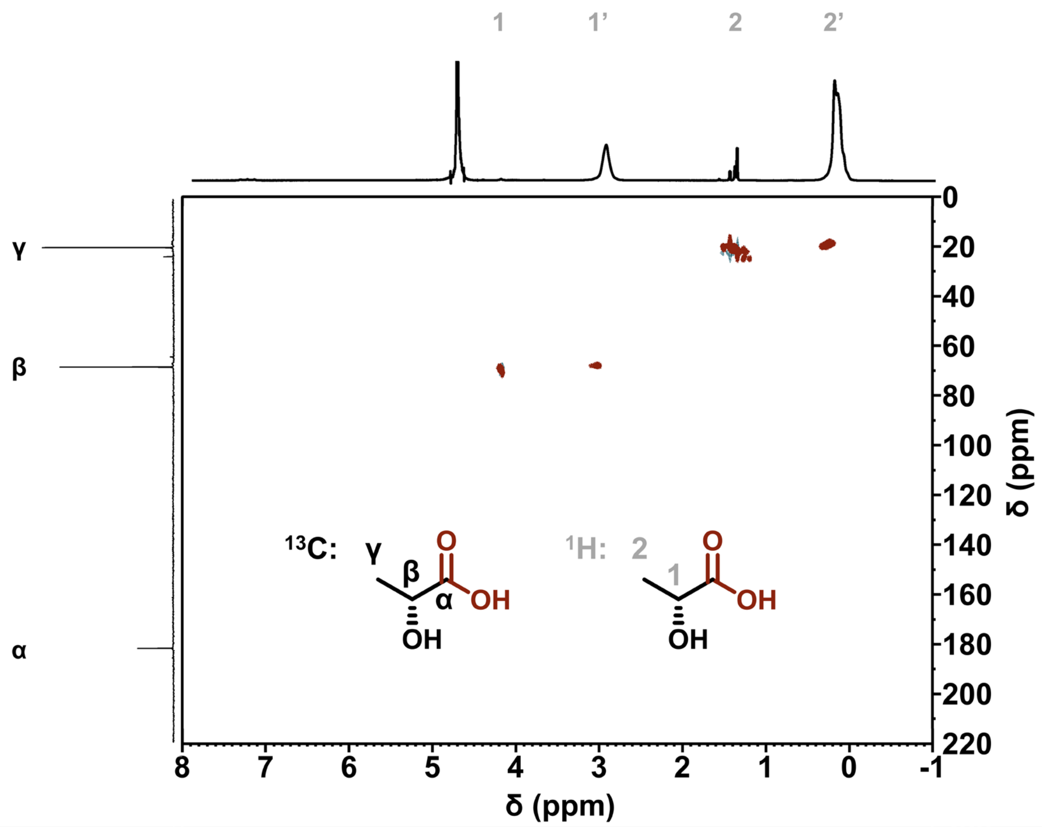

Figure 7: *^{1}H-^{13}C multiplicity-edited HSQC 2D NMR spectrum of {Mo_{132}(($_R$)-Lactate)$_{30}$} in D_2O. The CH and CH_3 signals are highlighted as red. The spectrum confirms the correlation between peaks of the free (R)-lactic acid and their related encapsulated peak signals, in both the ^{13}C and ^{1}H spectra.*

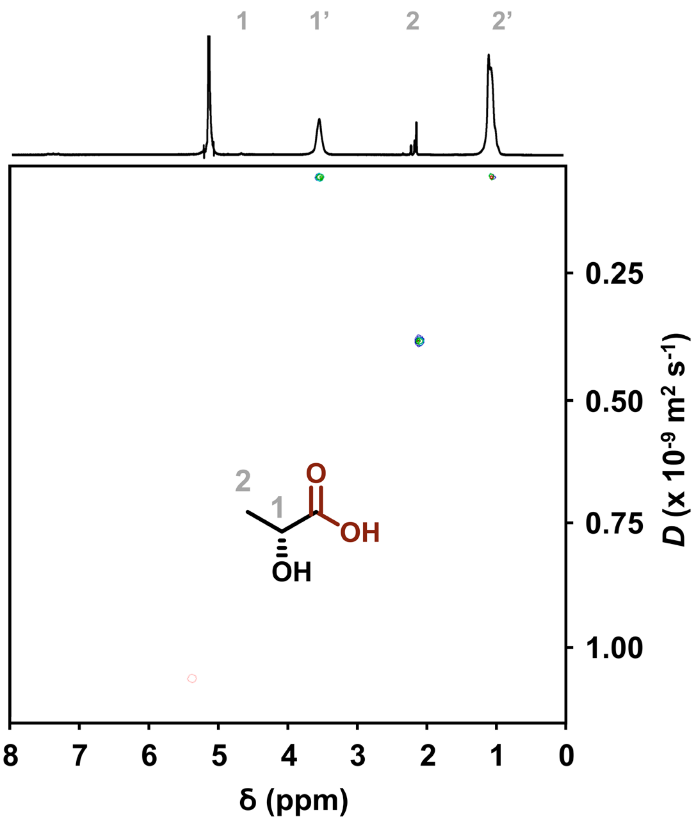

Figure 8: *DOSY NMR spectrum of {Mo_{132}((R)-Lactate)$_{30}$}.*

Chiral Recognition by $\{Mo_{132}(Lactate)_{30}\}$ Host Pores via ^{1}H NMR

<u>Amino Alcohol Guest Selection</u>

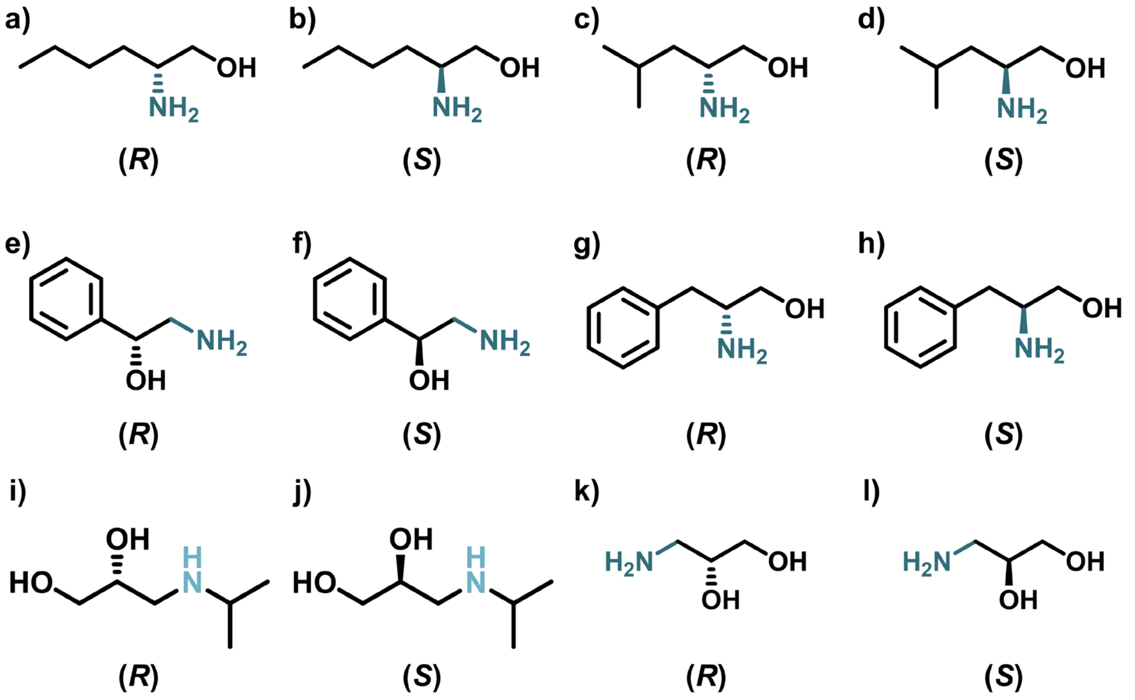

Figure 9: *Structures of selected guests to probe $\{Mo_2\}$-formed pore interactions. The amine group, which is required for pore interaction, is highlighted in light blue. a) (R)-2-amino-1-hexanol , b) (S)-2-amino-1-hexanol, c) (R)-leucinol, d) (S)-leucinol, e) (R)-2-amino-1-phenylethanol, f) (S)-2-amino-1-phenylethanol, g) (R)-phenylalaninol, h) (S)-phenylalaninol, i) (R)-3-isopropylamino-1,2-propanediol, j) (S)-3-isopropylamino-1,2-propanediol, k) (R)-3-amino-1,2-propanediol, and l) (S)-3-amino-1,2-propanediol.*

<u>Scheme of Shift Separation Assignment</u>

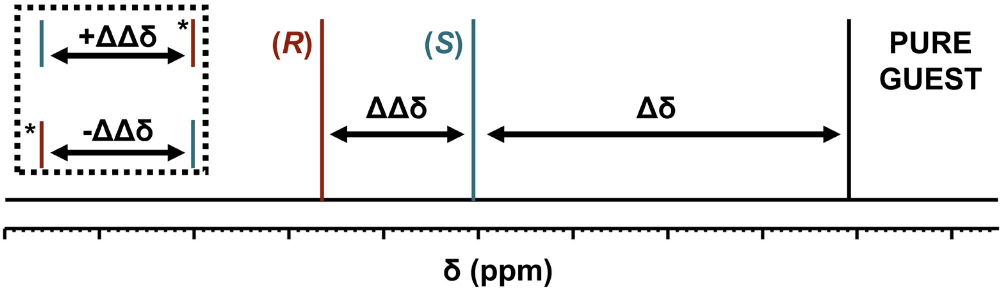

Figure 10: *Illustrative scheme of peak shifts (Δδ) of guest isomers in solutions containing interacting species (red and blue peaks) relative to the guest in D_2O only (black peak). The inset shows the relative sign given to the peak separation between the two guest isomers given by the relationship between the positions of the (R) and (S) isomers relative to one another (ΔΔδ).*

Control Reactions

Enantiopure Phenylalaninol with Enantiopure Lactic Acids

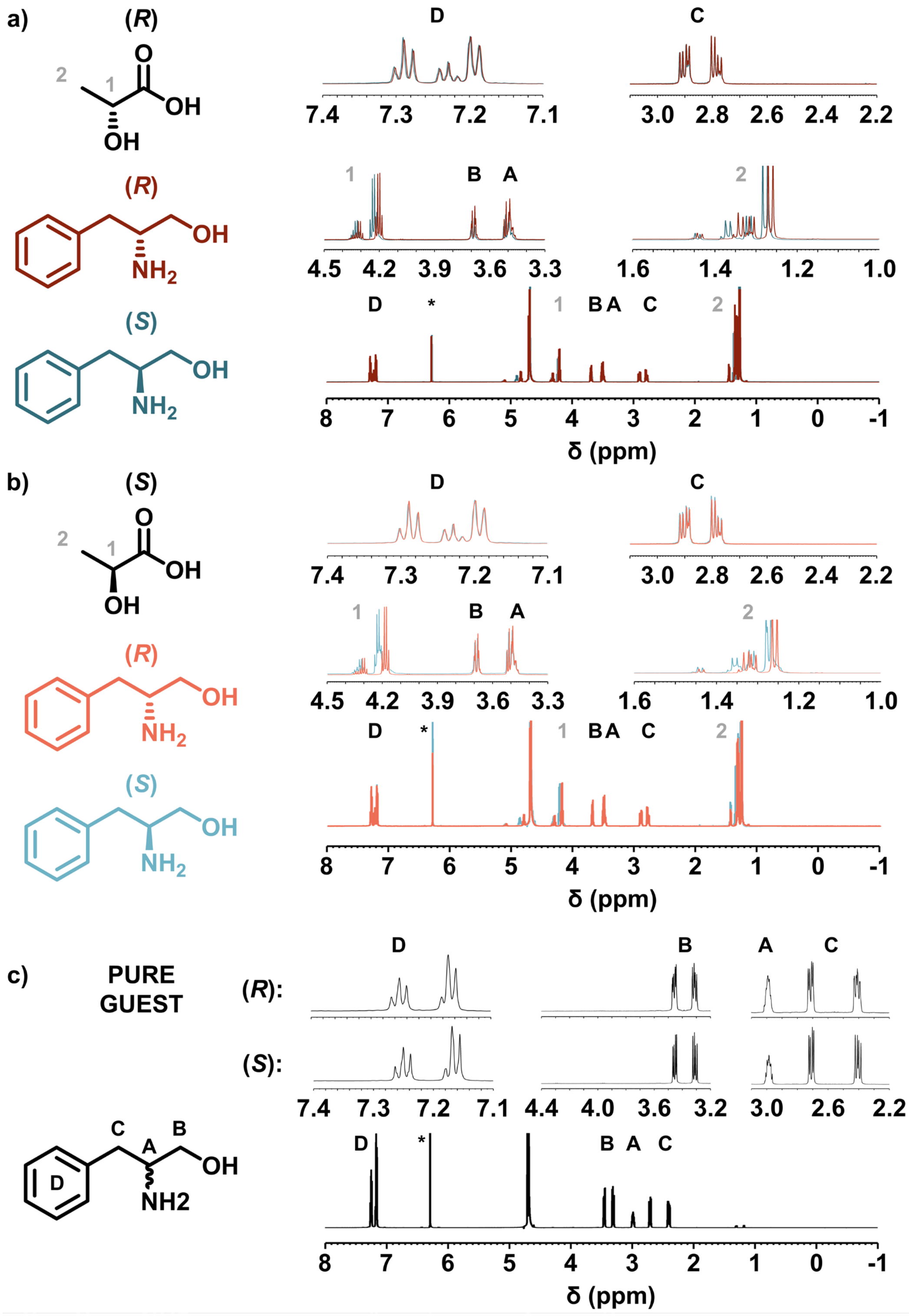

Figure 11: *^{1}H NMR of control reactions for phenylalaninol in D_2O. a) (R)-lactic acid with (R)-phenylalaninol (red), (S)-phenylalaninol (blue). b) (S)-lactic acid with (R)-phenylalaninol (red), and (S)-phenylalaninol (blue). c) (R)-phenylalaninol only.*

Enantiopure Phenylalaninol with $\{Mo_{132}(SO_4)_{30}\}$

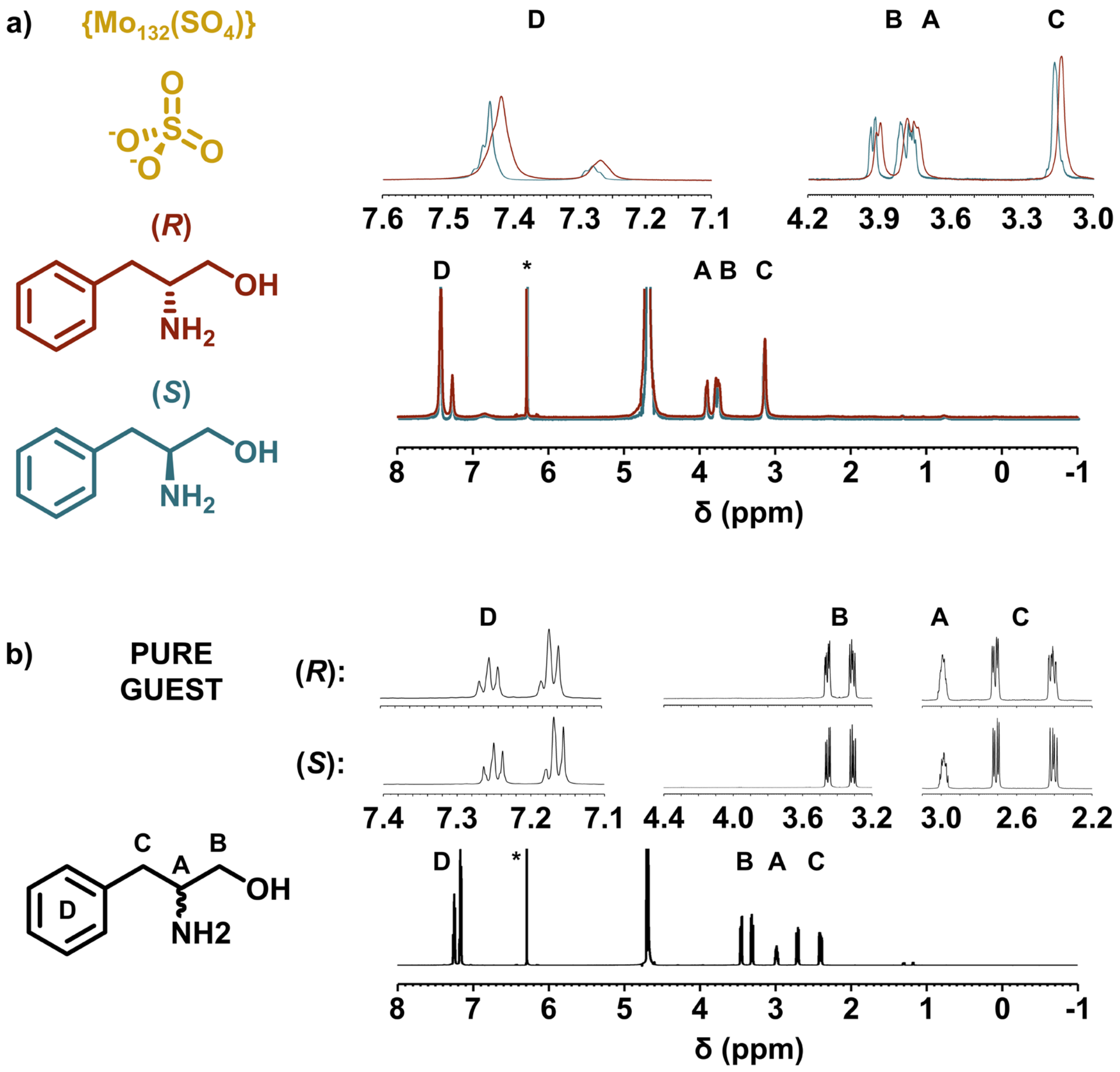

Figure 12: *^1H NMR of control reactions in D_2O of a) (R)-phenylalaninol (red) and (S)-phenylalaninol (blue) with $\{Mo_{132}(SO_4)_{30}\}$, showing no reversibility but slight peak separation, and b) (R)-phenylalaninol only, with the inset displaying select (S)-phenylalaninol spectrum sections.*

2-Dimethylaminoethanol with $\{Mo_{132}(SO_4)_{30}\}$ and Enantiopure $\{Mo_{132}(Lactate)_{30}\}$ – Non-chiral Guest Control Reaction

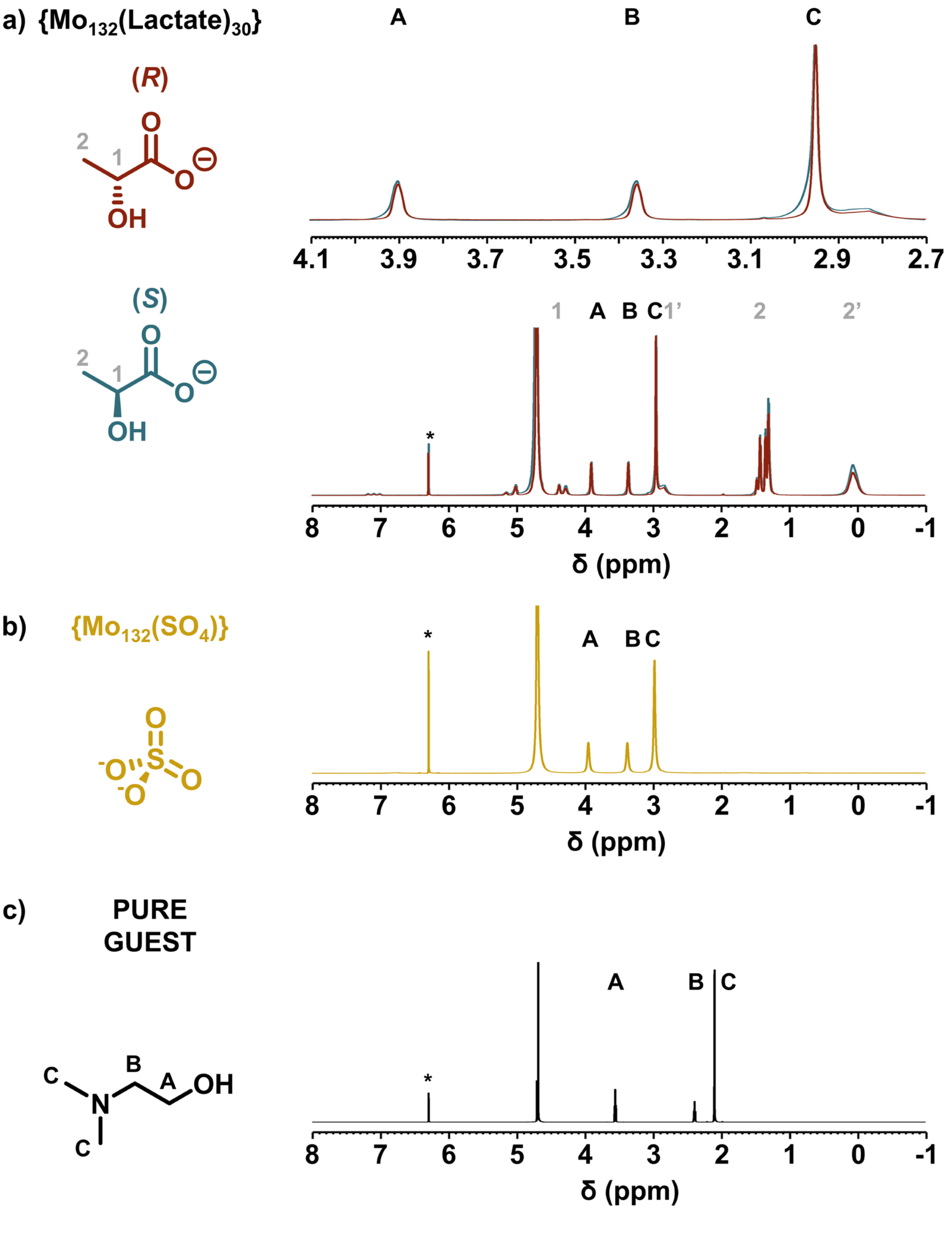

Figure 13: *^{1}H NMR of 2-dimethylaminoethanol with a) $\{Mo_{132}((R)\text{-}Lactate)_{30}\}$ (red) and $\{Mo_{132}((S)\text{-}Lactate)_{30}\}$ (blue), b) $\{Mo_{132}(SO_4)_{30}\}$, and c), the guest species only, all in D_2O.*

Separation of Enantiopure Amino Alcohol Guests with Enantiopure {Mo_{132}(Lactate)$_{30}$} Host Structures

Enantiopure Phenylalaninol with Enantiopure {Mo_{132}(Lactate)$_{30}$}

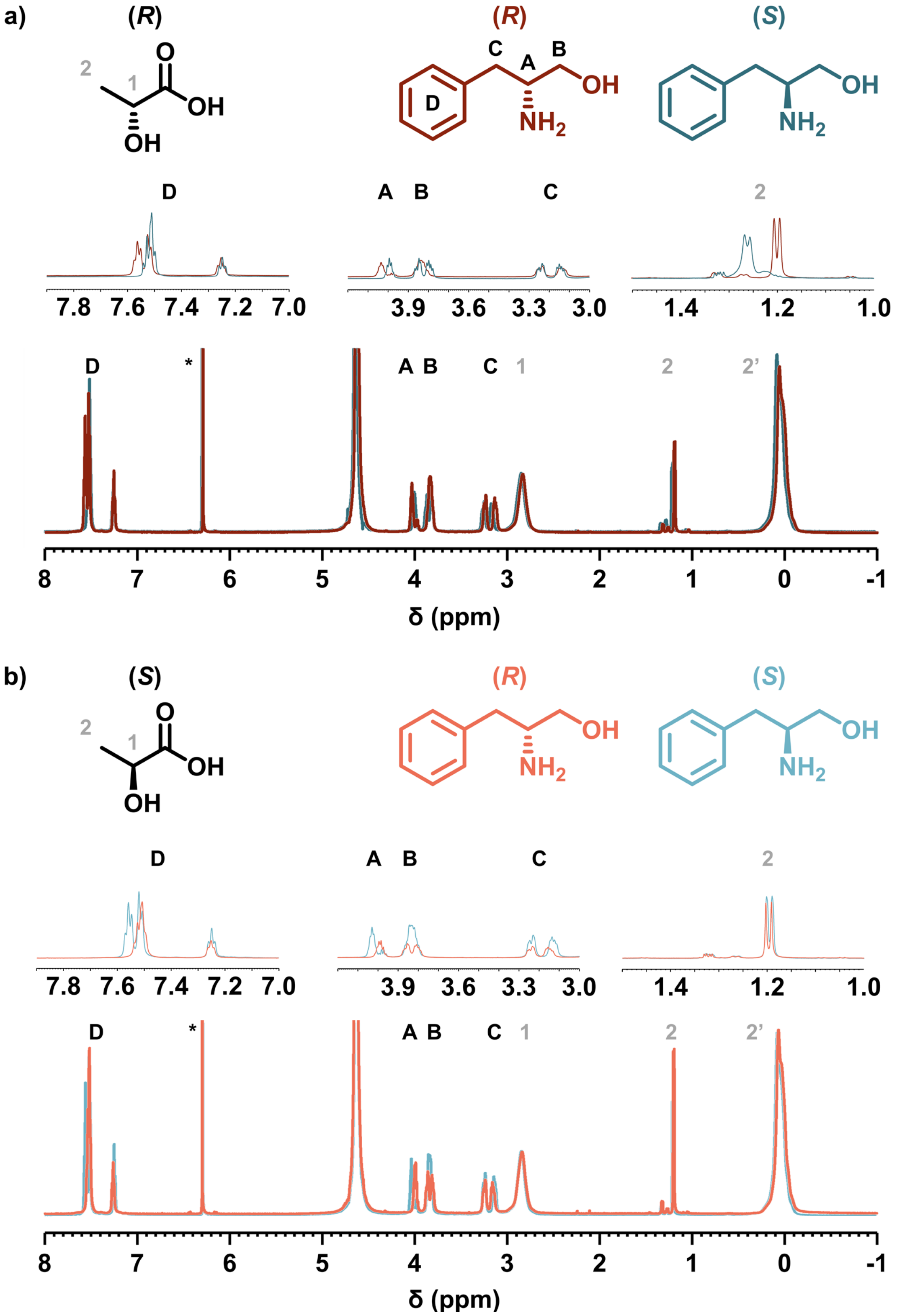

Figure 14: *^{1}H NMR of (R)-phenylalaninol (red/light red) and (S)-phenylalaninol (blue/light blue) guests with a) {Mo_{132}((R)-Lactate)$_{30}$}, and b) {Mo_{132}((S)-Lactate)$_{30}$}.*

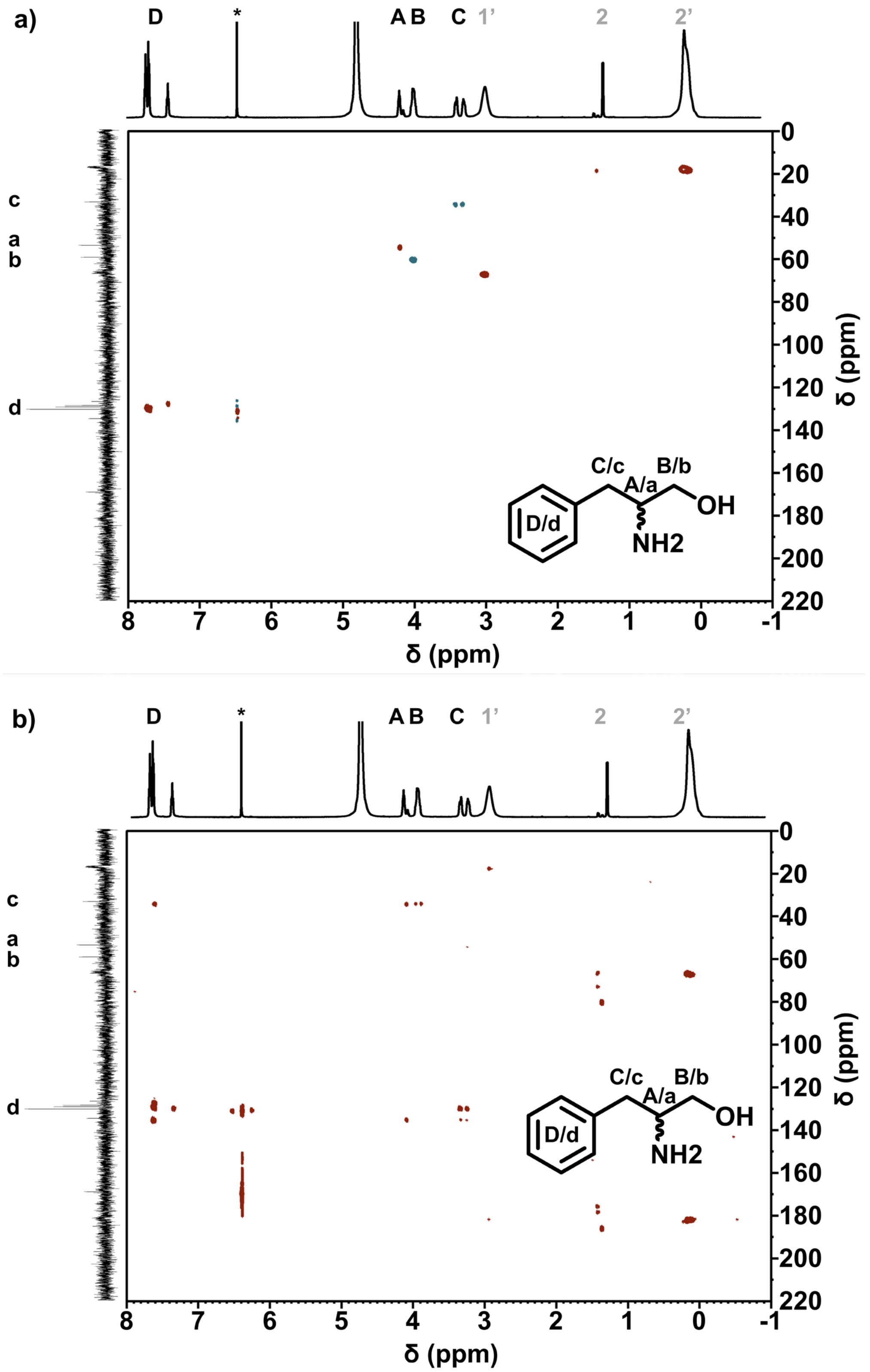

Figure 15: *a) HSQC, and b) HMBC spectra for phenylalaninol with {Mo_{132}((R)-Lactate)$_{30}$} in D_2O.*

Enantiopure 3-Amino-1,2-Propanediol with Enantiopure {Mo_{132}(Lactate)$_{30}$}

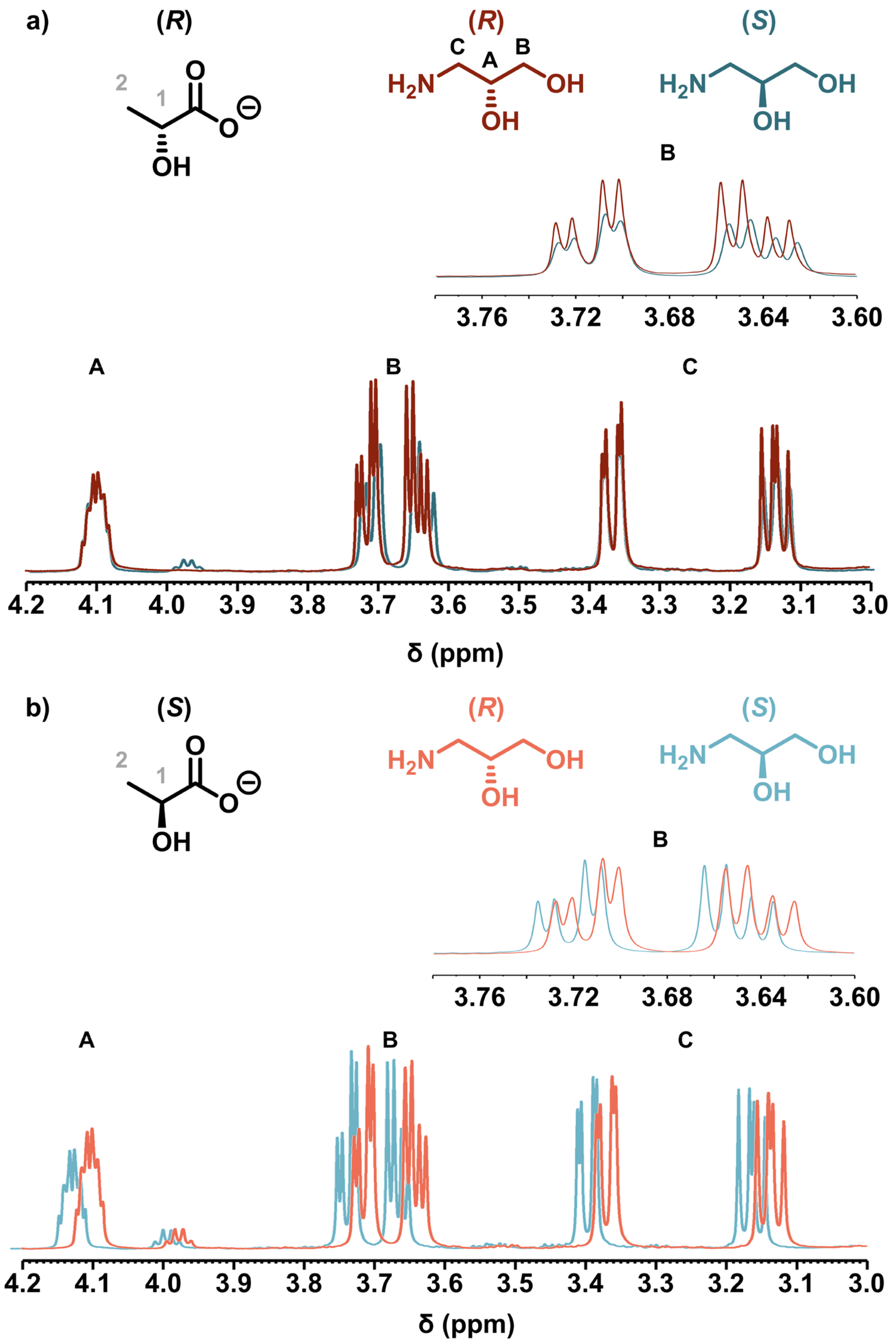

Figure 16: *^{1}H NMR of (R)-3-amino-1,2-propanediol (red/light red) and (S)-3-amino-1,2-propanediol (blue/light blue) guests with a) {Mo_{132}((R)-Lactate)$_{30}$}, and b) {Mo_{132}((S)-Lactate)$_{30}$}.*

Enantiopure Phenylglycinol with Enantiopure {Mo_{132}(Lactate)$_{30}$}

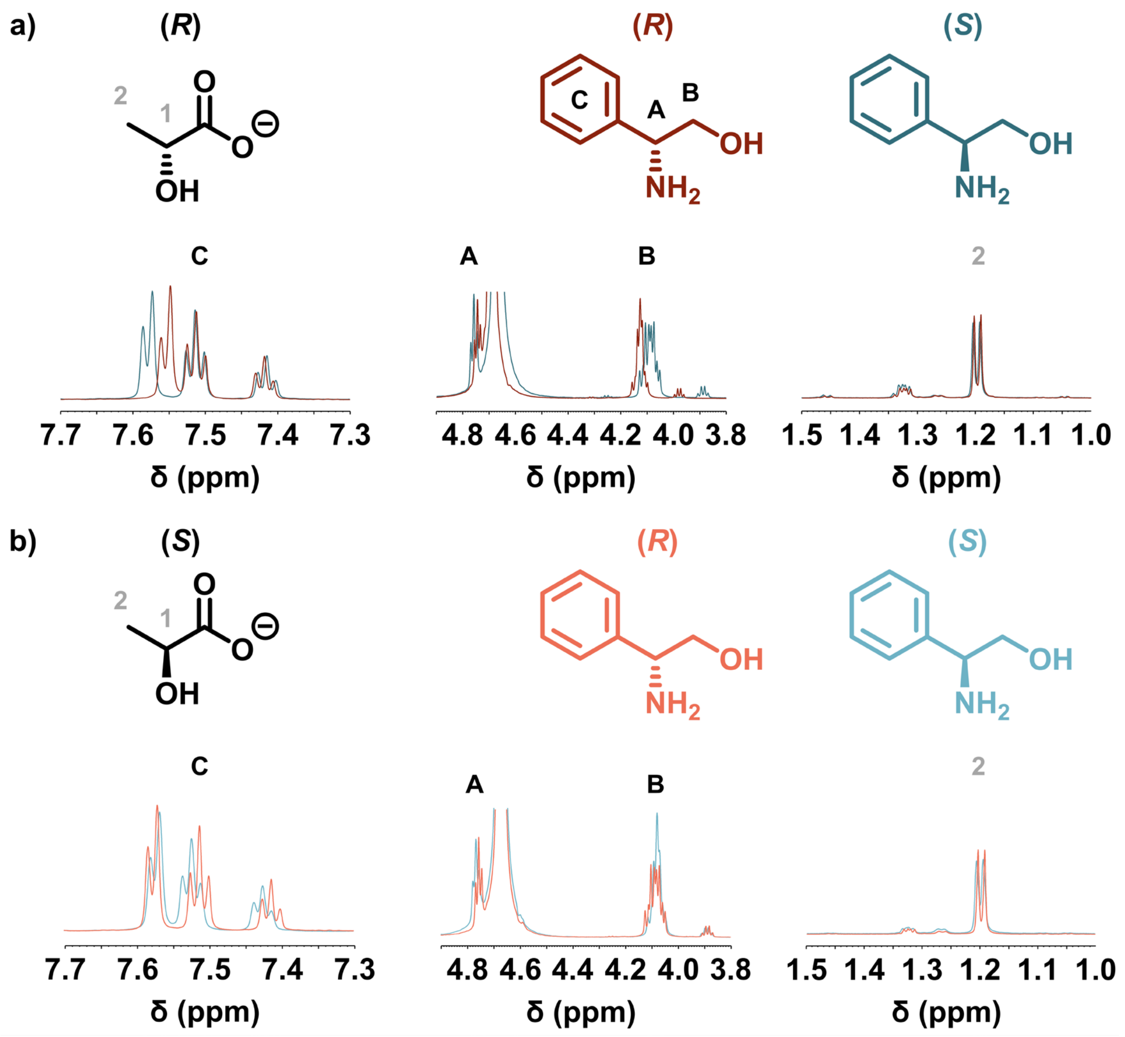

Figure 17: *^{1}H NMR of (R)-phenylglycinol (red/light red) and (S)-phenylglycinol (blue/light blue) guests with a) {Mo_{132}((R)-Lactate)$_{30}$}, and b) {Mo_{132}((S)-Lactate)$_{30}$}.*

Enantiopure 2-Amino-1-Phenylethanol with Enantiopure {Mo_{132}(Lactate)$_{30}$}

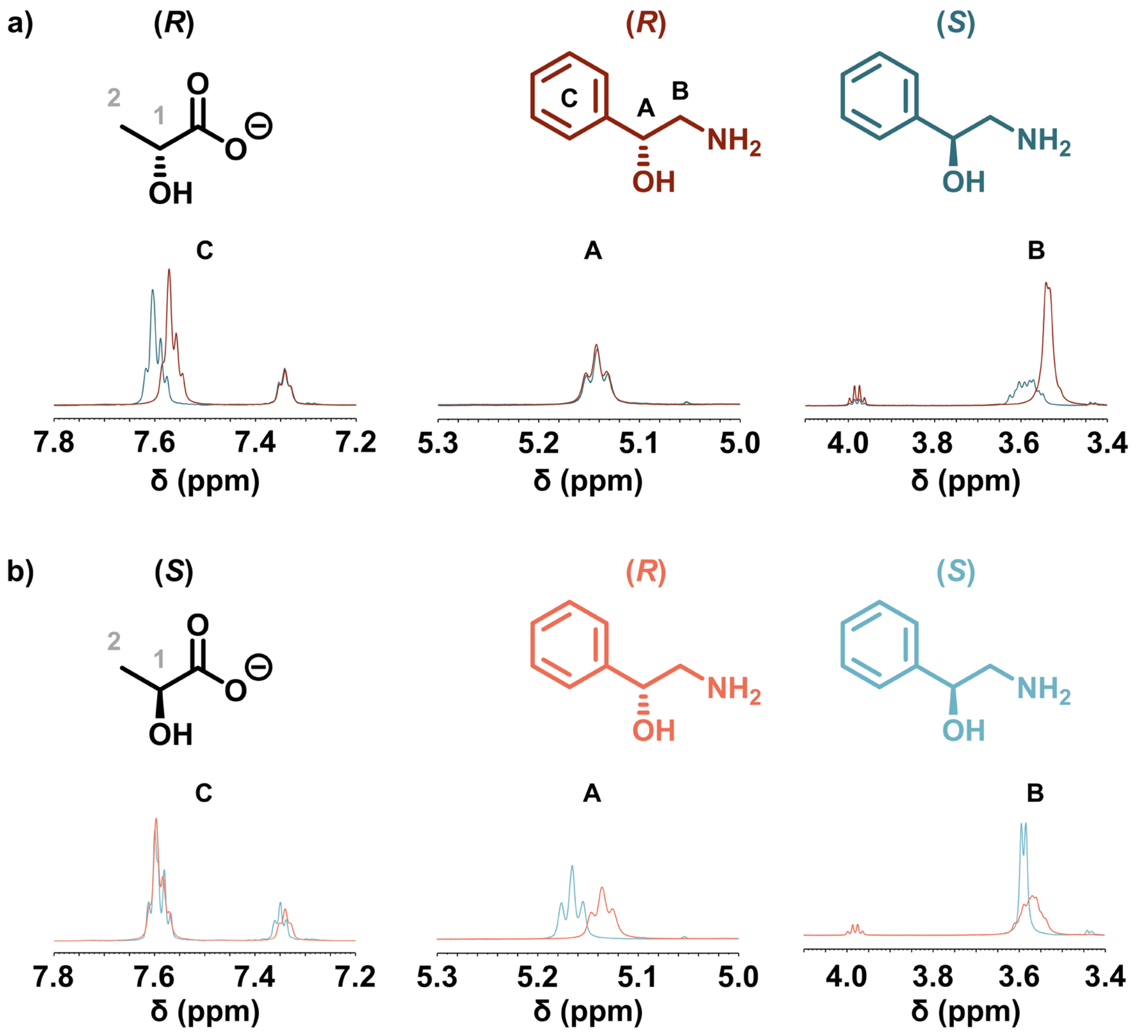

Figure 18: *^{1}H NMR of (R)-2-amino-1-phenylethanol (red) and (S)-2-amino-1-phenylethanol (blue) guests with a) {Mo_{132}((R)-Lactate)$_{30}$}, and b) {Mo_{132}((S)-Lactate)$_{30}$}.*

Separation of Racemic Amino Alcohol Guests with Enantiopure $\{Mo_{132}(Lactate)_{30}\}$ Host Structures

Racemic Phenylalaninol with Enantiopure $\{Mo_{132}(Lactate)_{30}\}$

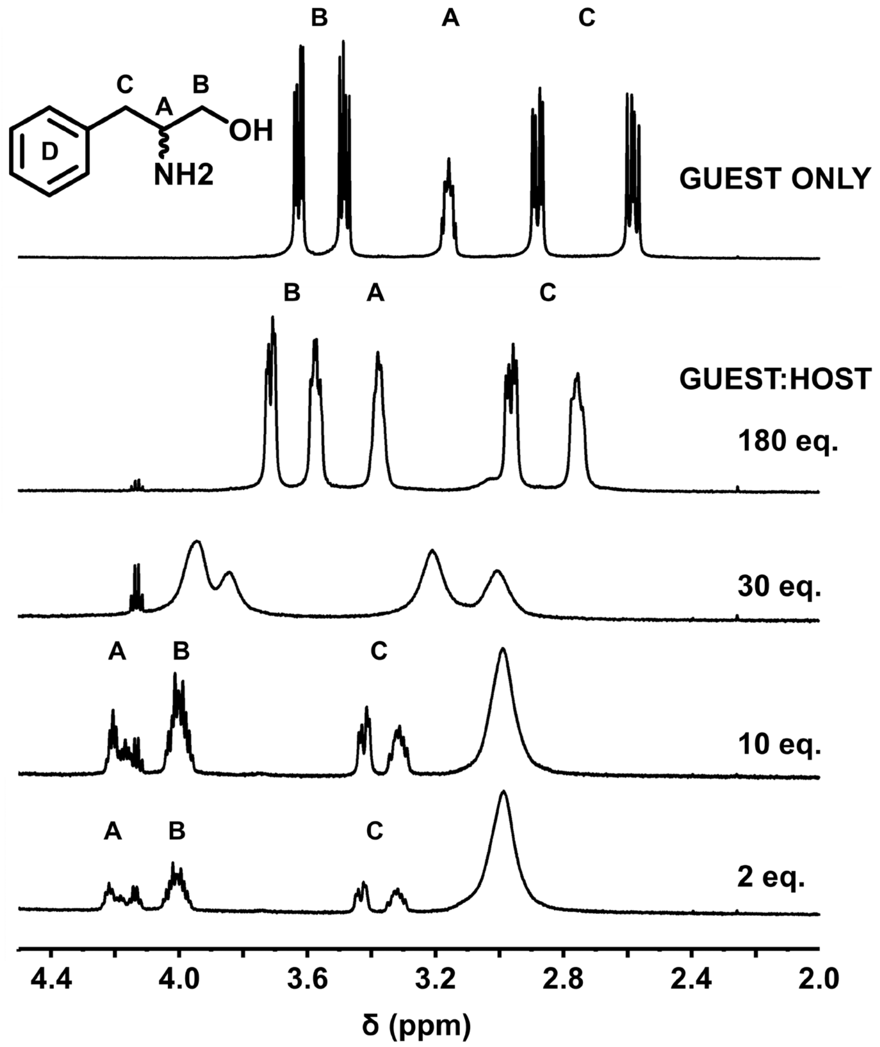

Figure 19: *Spectra of racemic (R/S)-phenylalaninol only (top), and as various added equivalents with respect to the $\{Mo_{132}((R)\text{-}Lactate)_{30}\}$ host. At 30 equivalents the features of the spectrum are lost as the $\{Mo_{132}((R)\text{-}Lactate)_{30}\}$ structure begins to precipitate from the solution.*

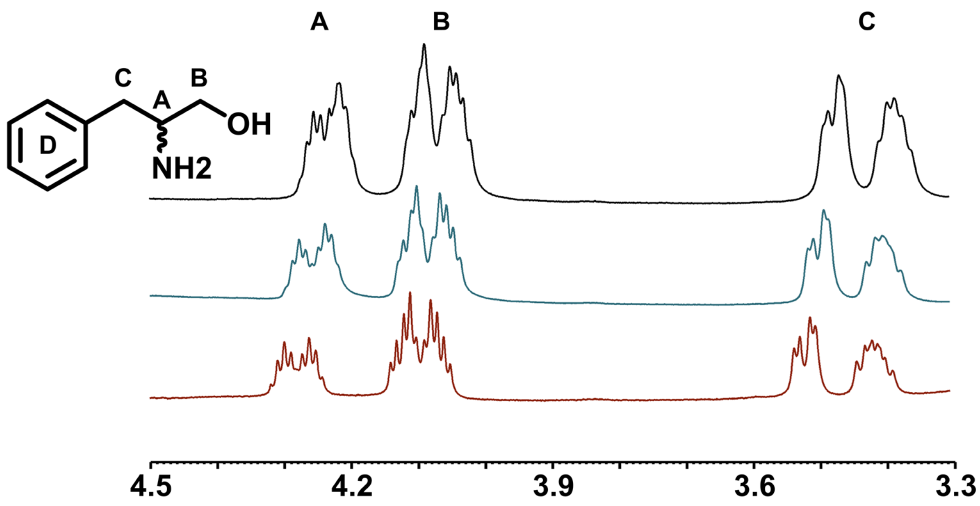

Figure 20: *Spectra of racemic (R/S)-phenylalaninol as various added equivalents (top – 9 eq., middle – 6 eq., bottom – 3 eq.) with respect to the {Mo_{132}((R)-Lactate)$_{30}$} host. Only a slight change in separation is observed for the ammonium groups (A) of each guest isomer, approximately 0.002 ppm, is observed.*

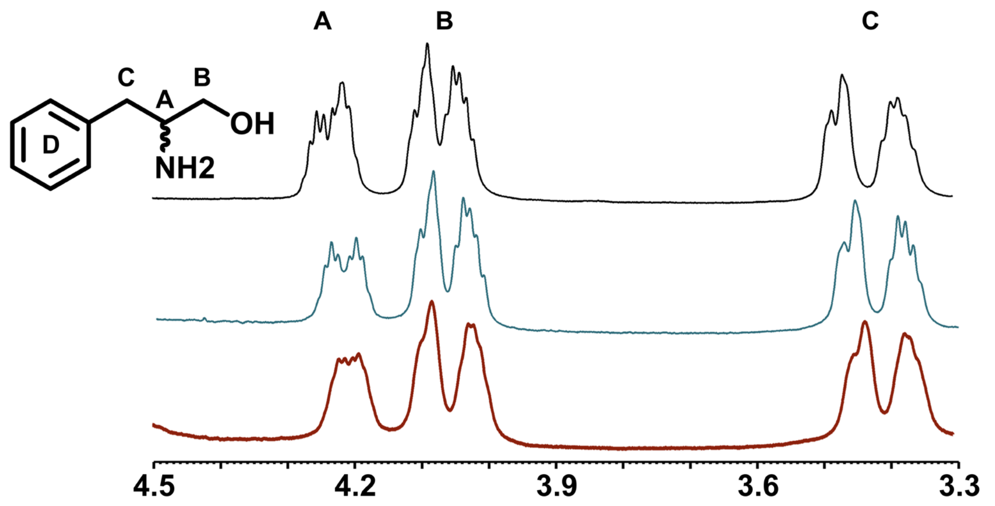

Figure 21: *Spectra of racemic (R/S)-phenylalaninol with {Mo_{132}((R)-Lactate)$_{30}$} at various pH values (top – 3, middle – 1.9, bottom – 1.1). Chemical shifts appear more upfield shifted due to a reduced interaction of the amino alcohol guests with the {Mo_{132}} host, due to the decreased coordination of lactate ligands at lower pH values.*

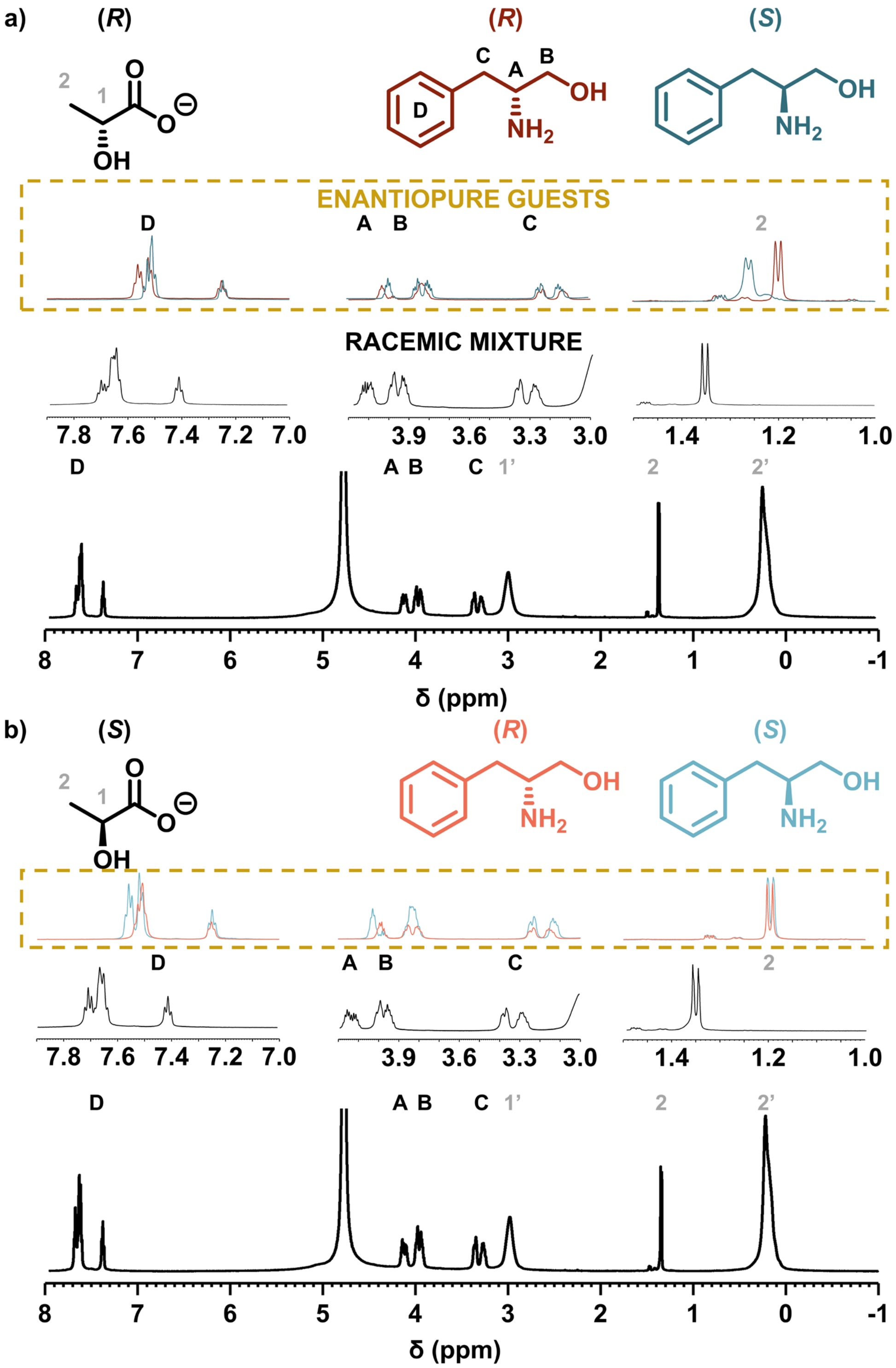

Figure 22: *Spectra of racemic (R/S)-phenylalaninol with a) {Mo_{132}((R)-Lactate)$_{30}$}, and b) {Mo_{132}((S)-Lactate)$_{30}$}. In each case, the spectra for the enantiopure (R)-phenylalaninol (red/light red) and (S)-phenylalaninol (blue/light blue) guests – in the dashed yellow boxes - with the relevant {Mo_{132}(Lactate)$_{30}$} host, is shown. The black spectra relate to the racemic guest mixtures. Insets are highlighted to show peaks of interest.*

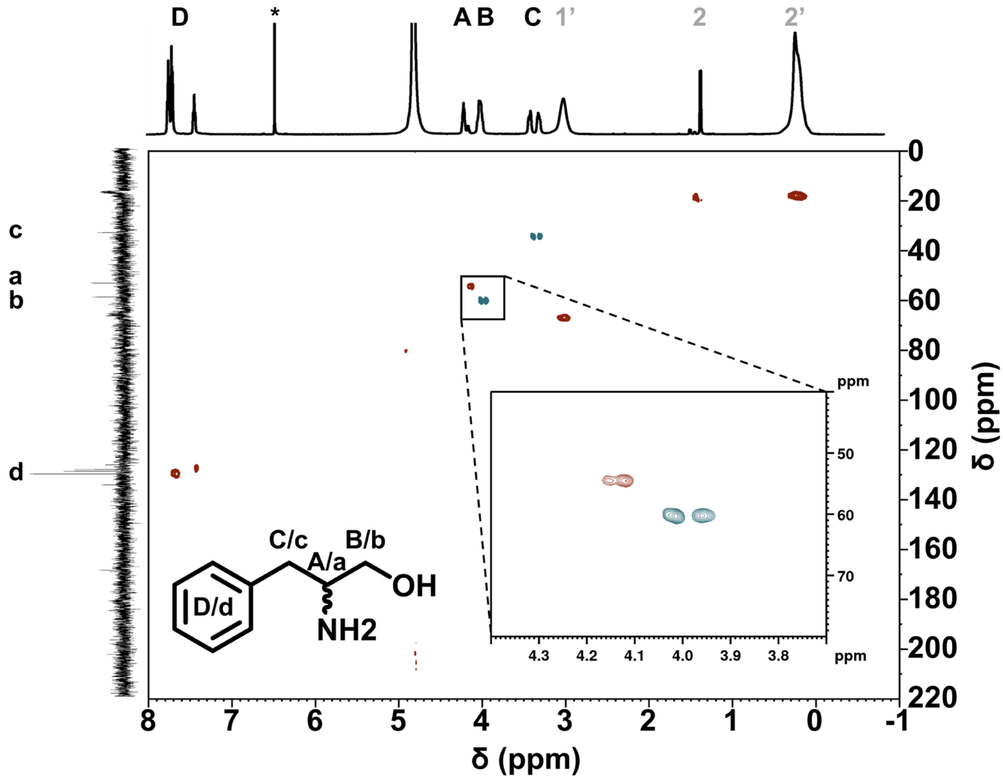

Figure 23: *300 K HSQC spectrum for (R/S)-phenylalaninol with {Mo_{132}((R)-Lactate)$_{30}$}, in D_2O. The inset relates to the highlighted peaks, CH(A) and CH_2(B).*

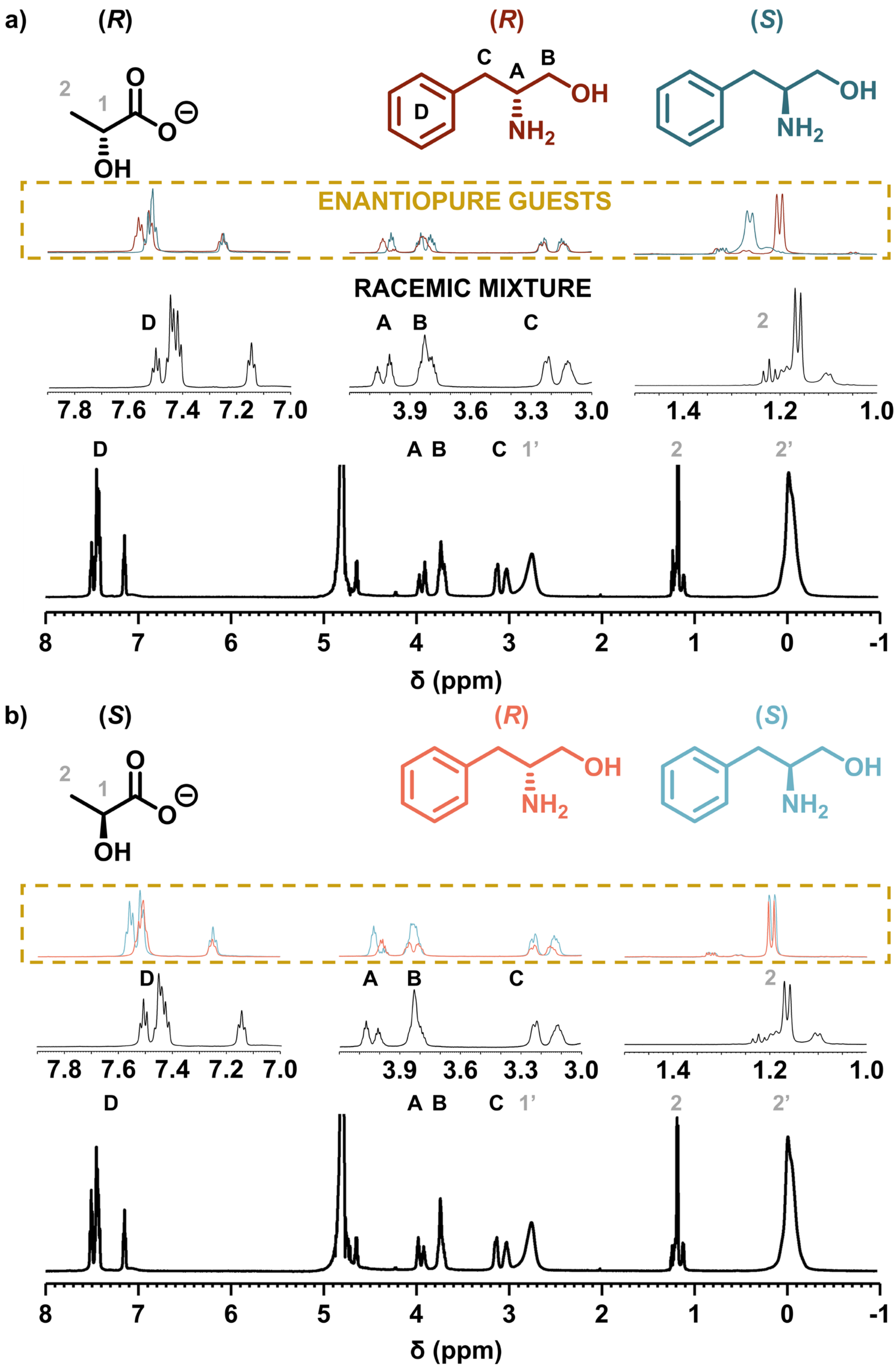

Figure 24: *Spectra of racemic (R/S)-phenylalaninol with a) {Mo_{132}((R)-Lactate)$_{30}$}, and b) {Mo_{132}((S)-Lactate)$_{30}$}, at 278 K. In each case, the spectra for the enantiopure (R)-phenylalaninol (red/light red) and (S)-phenylalaninol (blue/light blue) guests – in the dashed yellow boxes - with the relevant {Mo_{132}(Lactate)$_{30}$} host, is shown. The black spectra relate to the racemic guest mixtures. Insets are highlighted to show peaks of interest.*

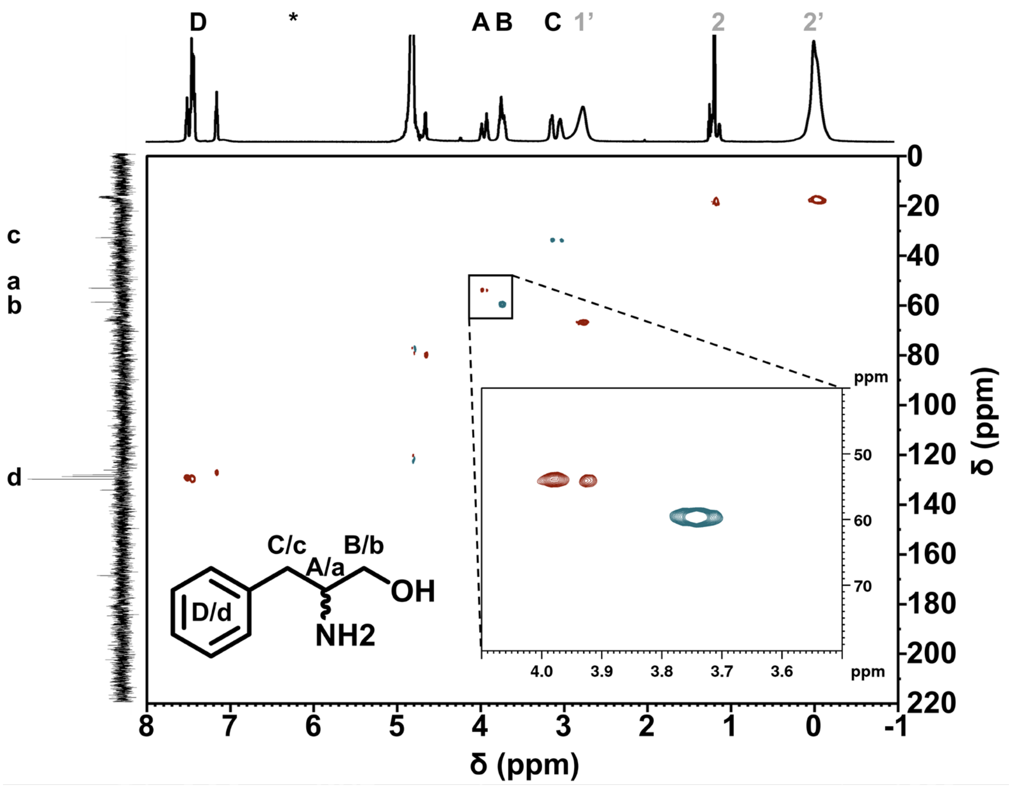

Figure 25: *Low temperature (278 K) HSQC spectrum for (R/S)-phenylalaninol with {Mo_{132}((R)-Lactate)$_{30}$}, in D_2O. The inset relates to the highlighted peaks, CH(A) and CH_2(B).*

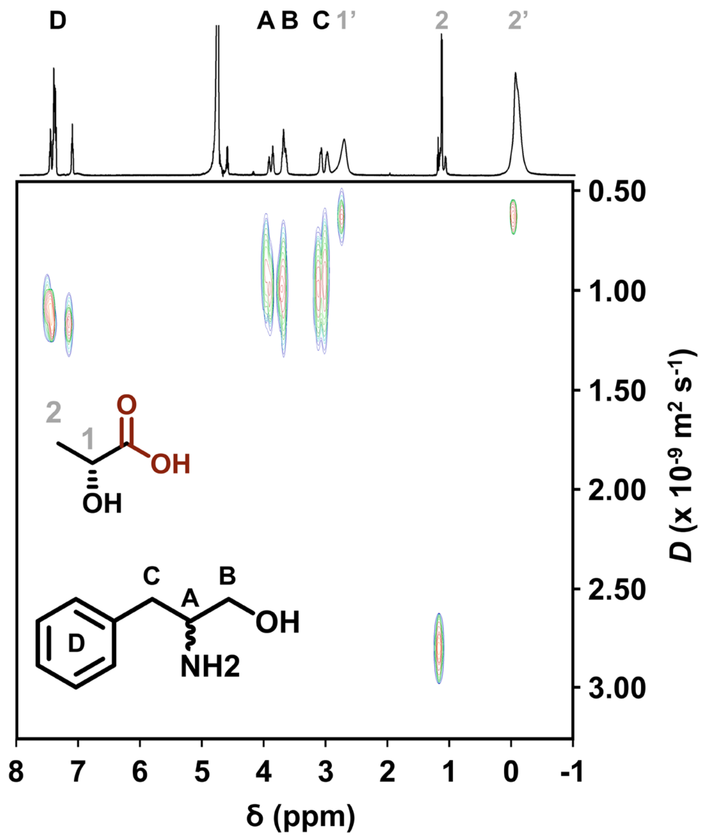

Figure 26: *DOSY NMR spectrum of {Mo_{132}((R)-Lactate)$_{30}$} and racemic (R/S)-phenylalaninol at 278 K.*

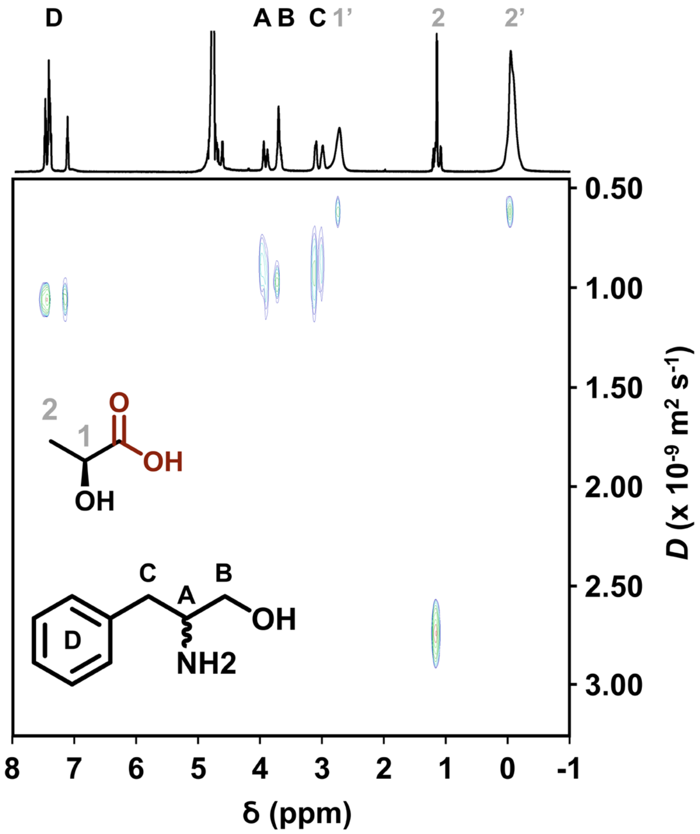

Figure 27: *DOSY NMR spectrum of {Mo_{132}((S)-Lactate)$_{30}$} and racemic (R/S)-phenylalaninol at 278 K.*

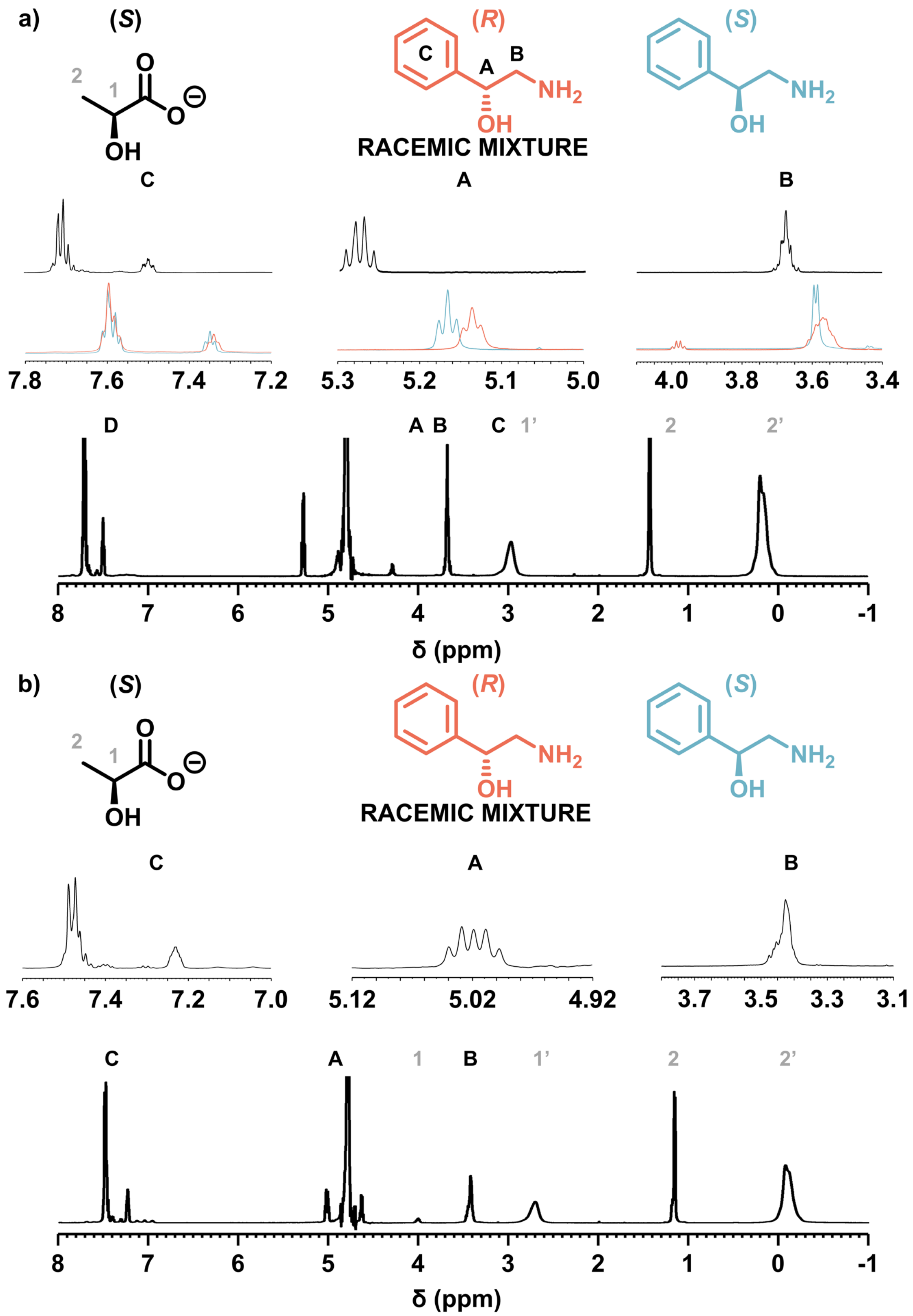

Figure 28: *^{1}H NMR of {Mo_{132}(S)-Lactate)$_{30}$} with racemic (R/S)- 2-amino-1-phenylethanol at a) 300 K, and b) 278 K.*

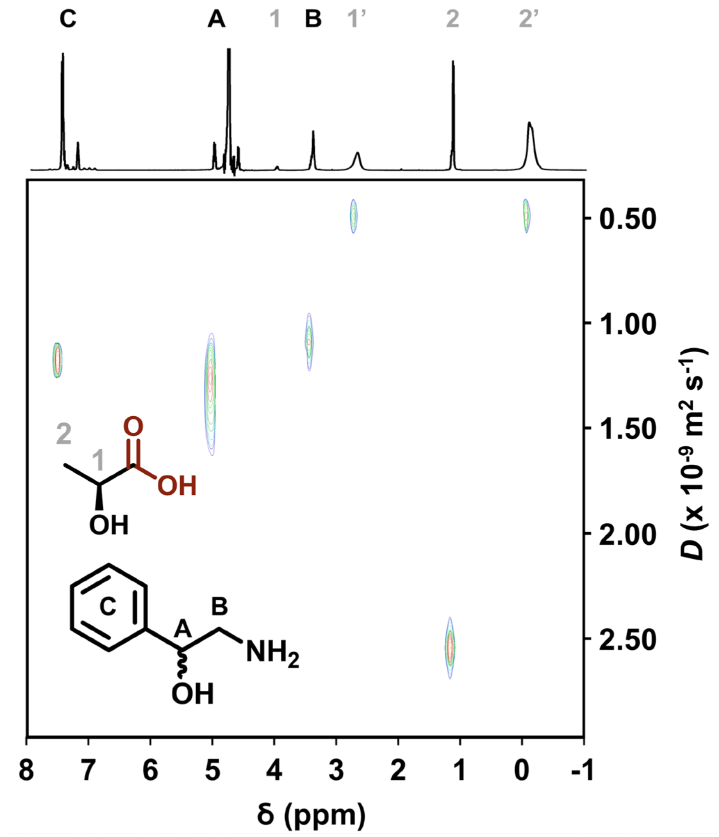

Figure 29: *DOSY NMR spectrum of {Mo_{132}((S)-Lactate)$_{30}$} and racemic (R/S)-2-amino-1-phenylethanol at 278 K.*

Separation of Scalemic Amino Alcohol Guests with Enantiopure $\{Mo_{132}(Lactate)_{30}\}$ Host Structures

Scalemic Phenylalaninol with Enantiopure $\{Mo_{132}(Lactate)_{30}\}$

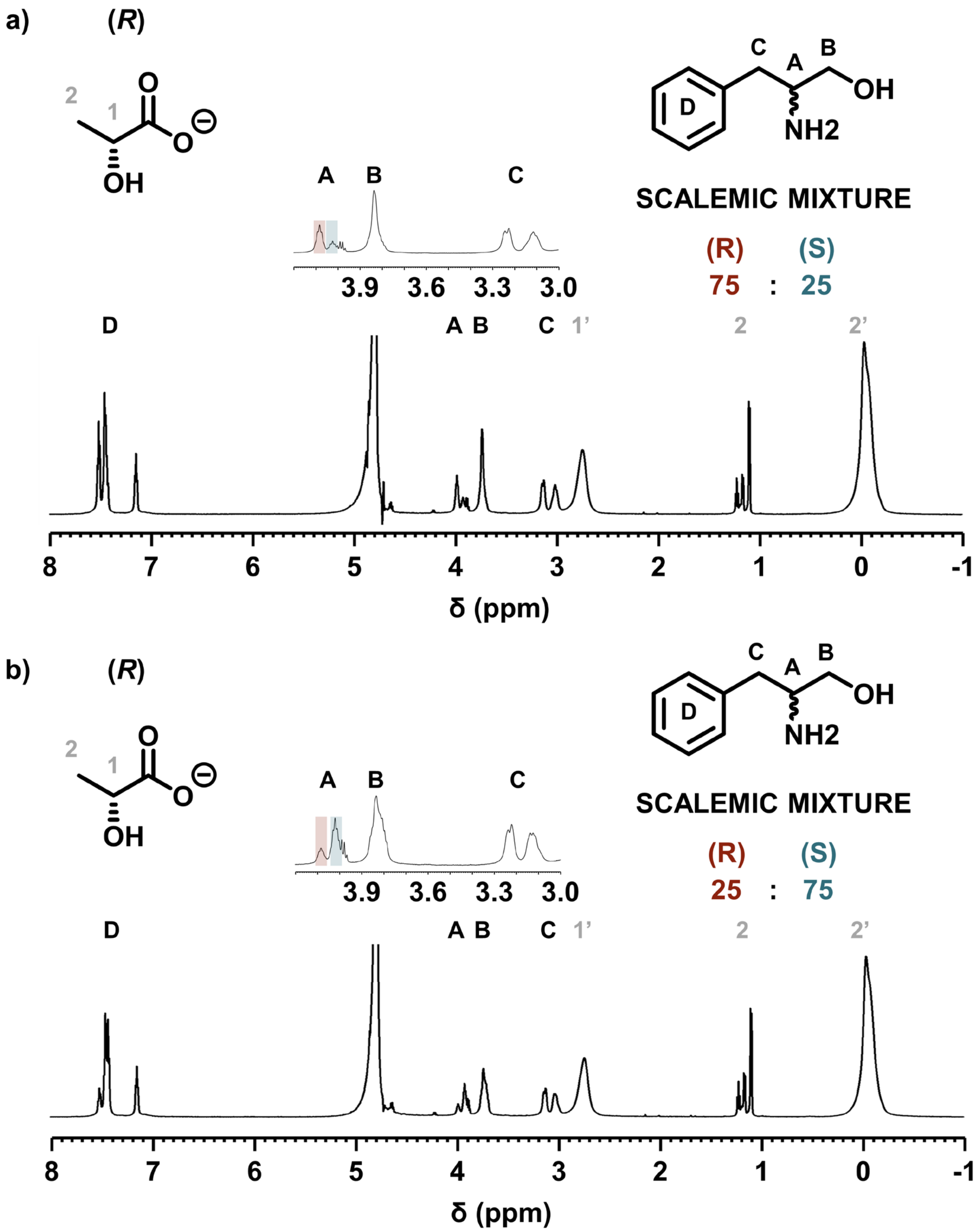

Figure 30: *^{1}H NMR spectra of the scalemic mixtures of (R) and (S)-phenylalaninol with $\{Mo_{132}((R)\text{-}Lactate)_{30}\}$ at 278 K. The ratio of (R) to (S)-phenylalaninol guests here is a) 75:25, and b) 25:75. The resulting effect on the CH(A) proton peak is highlighted for the (R) (red) and (S)-phenylalaninol (blue) guests.*

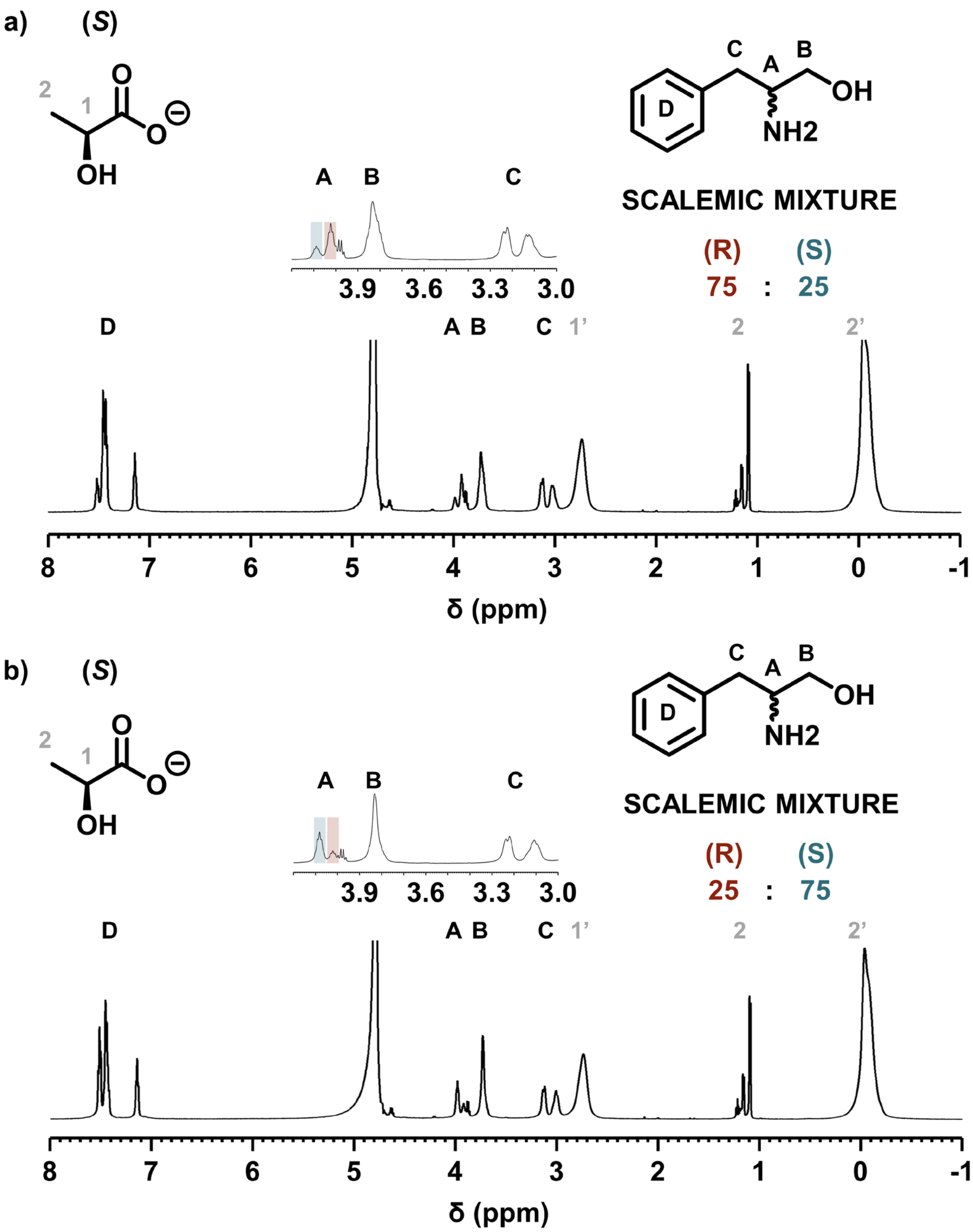

Figure 31: *^{1}H NMR spectra of the scalemic mixtures of (R) and (S)-phenylalaninol with {Mo_{132}((S)-Lactate)$_{30}$} at 278 K. The ratio of (R) to (S)-phenylalaninol guests here is a) 75:25, and b) 25:75. The resulting effect on the CH(A) proton peak is highlighted for the (R) (red) and (S)-phenylalaninol (blue) guests.*

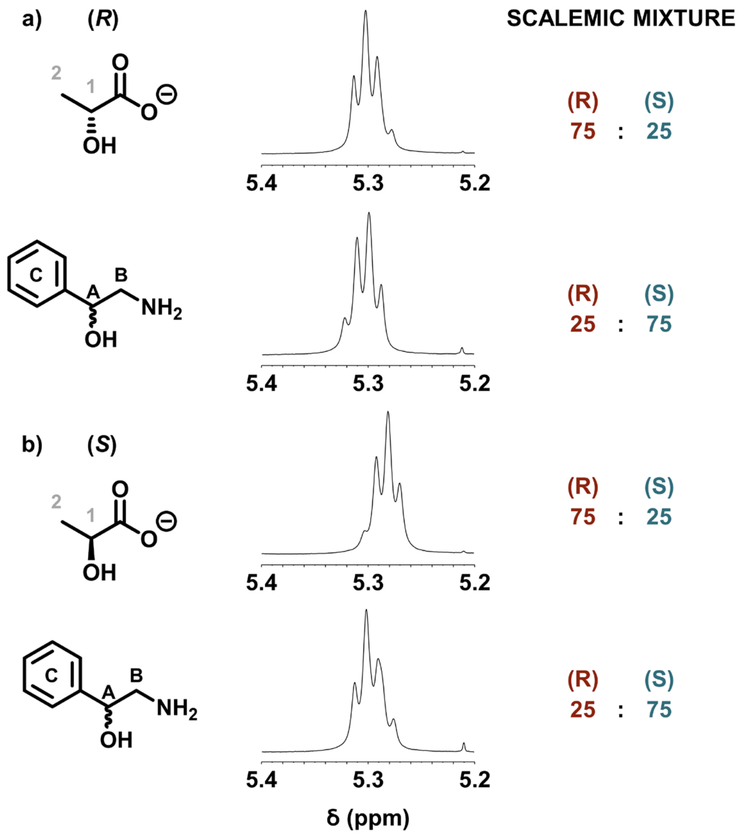

Figure 32: *Snapshot* [1]*H NMR spectra at 300 K of the scalemic mixtures of (R) and (S)-2-amino-1-phenylethanol with a) $\{Mo_{132}((R)\text{-}Lactate)_{30}\}$, and b) $\{Mo_{132}((S)\text{-}Lactate)_{30}\}$, highlighting the peak related to the proton located at the guest chiral centre (CH(A)). The ratio of (R) to (S)-2-amino-1-phenylethanol guests here is varied between 75:25 and 25:75.*

Determination of Association Constant of Amino Alcohols with $\{Mo_{132}(Lactate)_{30}\}$

For the determination of the strength of amino alcohol guest binding we have considered the $\{Mo_{132}\}$ pores individually, with twenty binding sites present on each molecule. Therefore, in determining the concentration of pores we have multiplied the concentration of $\{Mo_{132}\}$ by twenty. Diffusion NMR represents a fast, reliable method for the determination of association/dissociation constants. Dissociation constants (K_d) may be derived with a single experiment which produces diffusion coefficients for the pseudo-free guest (D_f), pseudo-bound guest (D_b), and the observed guest (D_o), which may be related to the total guest concentration (G_{TOT}) and host concentration (H_{TOT}) by Equation 1:

$$K_d = H_{TOT}\left(\frac{D_b - D_o}{D_o - D_f}\right) + G_{TOT}\left(\frac{D_o - D_b}{D_b - D_f}\right)$$

(*Equation 1*)

Equation 1 is subsequently used to determine association constants (K_a) for the guest species by Equation 2:

$$K_a = \frac{1}{K_d}$$

(*Equation 2*)

Table 1: *Peak positions (δ) and their related diffusion coefficients (D) used for the calculation of association constants (K_a) of amino alcohol guests with {Mo_{132}(Lactate)$_{30}$} host structures, at 278 K. The observed diffusion coefficients (D_o) related to the (R) or (S) guests are highlighted in red and blue, respectively.*

			**Diffusion Coefficient, *D* (x 10^{-11} m^2 s^{-1})**				**Association Constant, K_a (M^{-1})**	
			**D_b**	**D_f**	**D_o**			
**{Mo_{132}} HOST**	**GUEST**	*δ (ppm)*	*0.00/ 2.74*	*1.18*	*3.95*	*3.89*	**(*R*)**	**(*S*)**
(*R*)-Lactate	Phenylalaninol		6.52	28.2	9.11	10.06	**350**	**190**
(*S*)-Lactate	Phenylalaninol		6.30	27.5	8.63	9.41	**390**	**270**
		δ (ppm)	*-0.08/ 2.72*	*1.16*	*5.04*	*5.00*	**(*R*)**	**(*S*)**
(*R*)-Lactate	2-Amino-1-phenylethanol		5.08	25.5	10.65	10.55	**110**	**110**
(*S*)-Lactate	2-Amino-1-phenylethanol		5.08	25.5	13.0	13.3	**55**	**60**

Free lactic acid is considered as a non-interacting molecule, due to the slow exchange mechanism this species undergoes from the external to the internal {Mo_{132}} environment. Therefore, in Table 1 the diffusion coefficient for the free guest is taken to be that of free lactic acid. A clear error in our calculation will therefore arise from differences in the diffusion coefficients of the free guest itself and the free lactic acid, which should be larger for the guest due to the larger size of this species. Additionally, the diffusion coefficient of the entirely bound guest is taken to be the value associated with the coordinated lactate ligands, as these represent the approximate overall value of the {Mo_{132}} structure itself. Although the obtained association/dissociation constants are presented related to the average value of an individual pore site with a guest binding in a 1:1 ratio, allosteric effects are likely to effect individual pore binding abilities, due to sharing of coordinated ligands between two pore sites. An experimental determination of the binding stoichiometry was performed, using the method of continuous variation (Job's method), however data loss in the form of broadened NMR peaks at guest ratios greater than 1:1 hindered assignment via this method. This method did indicate that at least a 1:1 host:guest ratio is occurring.
